# Pharmacological inhibition of LSD1 suppresses growth of hepatocellular carcinoma by inducing GADD45B

**DOI:** 10.1002/mco2.269

**Published:** 2023-05-24

**Authors:** Na Sang, Xi Zhong, Kun Gou, Huan Liu, Jing Xu, Yang Zhou, Xia Zhou, Yuanzhi Liu, Zhiqian Chen, Yue Zhou, Yan Li, Lei Tao, Na Su, Lingyun Zhou, Jiahao Qiu, Xinyu Yang, Zeping Zuo, Li Fu, Jingyao Zhang, Dan Li, Cong Li, Qingxiang Sun, Jian Lei, Rui Li, Shengyong Yang, Xiaobo Cen, Yinglan Zhao

**Affiliations:** ^1^ Department of Biotherapy Cancer Center and State Key Laboratory of Biotherapy West China Hospital, West China Medical School, Sichuan University Chengdu China; ^2^ Department of Radiation Oncology Radiation Oncology Key Laboratory of Sichuan Province Sichuan Clinical Research Center for Cancer Sichuan Cancer Hospital & Institute, Sichuan Cancer Center, Affiliated Cancer Hospital of University of Electronic Science and Technology of China Chengdu China; ^3^ Department of Pharmacology Key Laboratory of Drug Targeting and Drug Delivery System of the Education Ministry Sichuan Engineering Laboratory for Plant‐Sourced Drug and Sichuan Research Center for Drug Precision Industrial Technology West China School of Pharmacy Sichuan University Chengdu China; ^4^ National Chengdu Center for Safety Evaluation of Drugs State Key Laboratory of Biotherapy West China Hospital Sichuan University Chengdu China; ^5^ Department of Pharmacy West China Hospital, West China Medical School, Sichuan University Chengdu China; ^6^ Center of Infectious Diseases West China Hospital Sichuan University Chengdu China; ^7^ Core Facility Center West China Hospital Sichuan University Chengdu China

**Keywords:** drug combination, GADD45B, hepatocellular carcinoma, LSD1 inhibitor

## Abstract

Lysine‐specific histone demethylase 1 (LSD1) is an attractive target for malignancies therapy. Nevertheless, its role in hepatocellular carcinoma (HCC) progression and the potential of its inhibitor in HCC therapy remains unclear. Here, we show that LSD1 overexpression in human HCC tissues is associated with HCC progression and poor patient survival. ZY0511, a highly selective and potent inhibitor of LSD1, suppressed human HCC cell proliferation in vitro and tumor growth in cell‐derived and patient‐derived HCC xenograft models in vivo. Mechanistically, ZY0511 induced mRNA expression of growth arrest and DNA damage‐inducible gene 45beta (*GADD45B*) by inducing histone H3 at lysine 4 (H3K4) methylation at the promoter of *GADD45B*, a novel target gene of LSD1. In human HCC tissues, LSD1 level was correlated with a decreased level of GADD45B, which was associated with HCC progression and predicted poor patient survival. Moreover, co‐administration of ZY0511 and DTP3, which specifically enhanced the pro‐apoptotic effect of GADD45B, effectively inhibited HCC cell proliferation both in vitro and in vivo. Collectively, our study revealed the potential value of LSD1 as a promising target of HCC therapy. ZY0511 is a promising candidate for HCC therapy through upregulating GADD45B, thereby providing a novel combinatorial strategy for treating HCC.

## Graphical abstract

In the present study, we demonstrated ZY0511 effectively inhibited hepatocellular carcinoma (HCC) cell proliferation by upregulating the expression of DNA damage‐inducible gene 45beta (*GADD45B*), a direct downstream target of lysine‐specific histone demethylase 1 (LSD1). Notably, a combination of ZY0511 with DTP3, which enhanced pro‐apoptosis functions of GADD45B, synergistically inhibited HCC cell proliferation both in vitro and in vivo. The figure was partly generated using Servier Medical Art, provided by Servier, licensed under a Creative Commons Attribution 3.0 unported license.

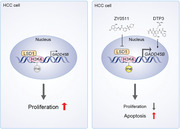

## 1 INTRODUCTION

Hepatocellular carcinoma (HCC), the most frequent primary liver cancer, is a highly aggressive solid tumor, ranking sixth in new‐diagnosis rate and third in cancer‐related death rate worldwide, with approximately 769,000 newly diagnosed patients and 705,000 mortalities per year.^1^, ^2^ Together with the recognition of its severity and harmfulness, great progress has been made in treatment. Non‐drug therapies, including hepatic resection, liver transplantation, trans‐arterial chemoembolization, and ablation, are all beneficial for patients with early and mid‐stage HCC.^3,^ ^4^ However, for 50% of patients with advanced HCC, systemic pharmacological treatment is currently the most effective.^2^, ^3^, ^4^ Small‐molecule targeted drugs such as sorafenib and lenvatinib, together with monoclonal antibodies such as nivolumab, are mainly used for the systematic treatment of advanced HCC.^5^ Despite the fact that new promising targeted systemic therapy has shown measurable efficacy with an objective response rate and prolonged the life expectancy of patients with advanced HCC, the therapeutic outcome is still far from satisfactory, mainly due to limited efficiency, drug resistance, and side effects of the above drugs. There remains an unmet need for novel and effective therapeutic drugs for HCC therapy.

Alterations in the epigenetic signature play pivotal parts in tumorigenesis and progression, and targeting epigenetic dysregulations has become a promising strategy for cancer therapy.^6^ Until now, epidrugs including DNA methyltransferase (DNMT) inhibitor guadecitabine and histone deacetylase (HDAC) inhibitors vorinostat and belinostat were experimentally tested for treating HCC, but they were limited to the clinical trials and did not enter beyond phase II trials, owing to the safety problem.^7^ Thus, developing rational epigenetic inhibitors with better efficacy and safety profiling based on an in‐depth understanding of the epigenetics in HCC could be meaningful for improving the overall survival of HCC patients. Lysine‐specific histone demethylase 1 (LSD1, also known as KDM1A) is the first of lysine demethylases discovered.^8^ It is responsible for the demethylation of the mono‐ and dimethylated forms of H3K4 and histone H3 lysine 9 (H3K9), which ultimately regulates target genes expression.^8^, ^9^ LSD1 overexpression is observed in multiple human malignancies, including HCC, colorectal cancer (CRC), acute myeloid leukemia (AML), oral cancer, small cell lung cancer (SCLC), breast cancer, and prostate cancer, and is associated with poor patient survival rates.^10^ Several data highlighted LSD1 to be implicated within HCC progression by controlling cell proliferation and maintaining cancer stem cell self‐renewal, as well as glycolytic and mitochondrial metabolism although the role of LSD1 in HCC progression remained incompletely understood.^9^, ^11^, ^12^ Meanwhile, LSD1 knockdown by RNAi, CRISPR/Cas9 technology, or pharmacological inhibitors inhibited tumor proliferative, invasive, and migrative properties.^10^

Recently, several selected LSD1 inhibitors, including GSK‐2879552, ORY‐1001, and INCB059872 have been developed, and their clinical trials for treating malignancies have been in progress.^10^ These inhibitors mainly exhibited high potency against hematological tumors such as AML, myelodysplastic syndromes, and relapsed Ewing sarcoma but showed less effectiveness against solid tumors except for SCLC.^13^ Substantial research was exerted for exploring LSD1 inhibitors against solid tumors. CBB1003 exhibited anti‐CRC potency although its structure–activity relationships remain unclear.^14^ SP2509 blocked the proliferative properties within the Ewing sarcoma tissue, and SP2577, similar in composition to SP2509, has been undergoing clinical trials within Ewing sarcoma population (NCT03600649).^15^ A few LSD1 inhibitors were used in combination with small molecular multikinase inhibitors for treating unresectable HCC in clinical trials. SP2509 and tranylcypromine enhanced the anti‐proliferation or apoptotic effects of regorafenib against HCC cells.^16^ GSK2879552 and pargyline suppressed the stemness of sorafenib‐resistant HCC cells.^17^ Although the above studies suggested that LSD1 inhibitors exhibited potential for HCC treatment or enhanced sensitivity of small molecular multikinase inhibitors against unresectable HCC, their low efficiency hindered their further use for treating HCC.

The growth arrest and DNA damage‐inducible gene 45beta (GADD45B) is a multifunctional protein that belongs to the GADD45 family. It plays important roles in cell cycle arrest, DNA repair, cell senescence, and apoptosis by interplaying with cellular proteins in response to physiological or external stressors.^18^ The downregulation of GADD45B was observed in human HCC tissues and was significantly associated with tumor progression.^19^ Therefore, GADD45B has been identified as a potential molecular marker in HCC. However, the biological significance and therapeutic potential of GADD45B in HCC remain unclear.

Our previous study reported that ZY0511, a potent and novel small molecular LSD1 inhibitor, exhibited a strong anti‐tumor efficiency against CRC and cervical cancer both in vitro and in vivo and is well tolerated.^20^, ^21^, ^22^ Based on the research above, we hypothesized that LSD1 inhibitors ZY0511 might effectively inhibit HCC proliferation, which is worth to be investigated. In the present study, we demonstrated ZY0511 effectively inhibited HCC cell proliferation by upregulating the expression of *GADD45B*, a direct downstream target of LSD1. Notably, a combination of ZY0511 with DTP3, which enhanced pro‐apoptosis function of GADD45B, synergistically inhibited HCC cell proliferation both in vitro and in vivo. Our results reveal LSD1 as a promising target for HCC therapy and offer pre‐clinical proof of concept for ZY0511 alone or in combination with the GADD45B modulator for HCC treatment.

## 2 RESULTS

### 2.1 LSD1 is overexpressed in human HCC tissues and positively correlated with poor patient survival rate

The expression of *KDM1A* in liver HCC (LIHC) and other five common gastrointestinal cancers was analyzed based on The Cancer Genome Atlas (TCGA) database. Three commonly used indicators including different expression between cancer tissues and normal adjacent tissues (NATs), different expression in different grades, and survival rate suggested that *KDM1A* was an unfavorable gene in LIHC tissues, compared with other five common digestive tract tumors (Figures S1A‐L and S2A‐F). The results showed that *KDM1A* exhibited a high level of expression in LIHC tissues, compared with that in NATs (Figure S1A), and its expression increased along with the stages of tumor progression (Figure S1D). The Kaplan–Meier analysis showed that the expression of *KDM1A* in HCC tissues was inversely correlated with the survival rate of patients (Figure S2A). In colon adenocarcinoma (COAD), rectum adenocarcinoma (READ), stomach adenocarcinoma (STAD), and esophageal carcinoma (ESCA), *KDM1A* expression was upregulated in cancer tissues, compared with that in NATs (Figure S1B,C,G,H), and slight difference was observed among different stages of cancers (Figure S1E,F,J,K). However, there was no correlation between *KDM1A* expression and survival rate in patients of COAD, READ, STAD, and ESCA (Figure S2B,C,D,E, respectively). The expression of *KDM1A* was significantly upregulated in some stages of pancreatic adenocarcinoma (PAAD; Figure S1L) and associated with poor survival rate of PAAD patients (Figure S2F), but no significant difference was observed in *KDM1A* expression between PAAD tissues and NATs (Figure S1I). Taken together, these data suggested that *KDM1A* expression is elevated in LIHC tissues and tightly links to LIHC development.

### 2.2 ZY0511 inhibits the proliferation of human HCC cells in vitro

**FIGURE 1:**
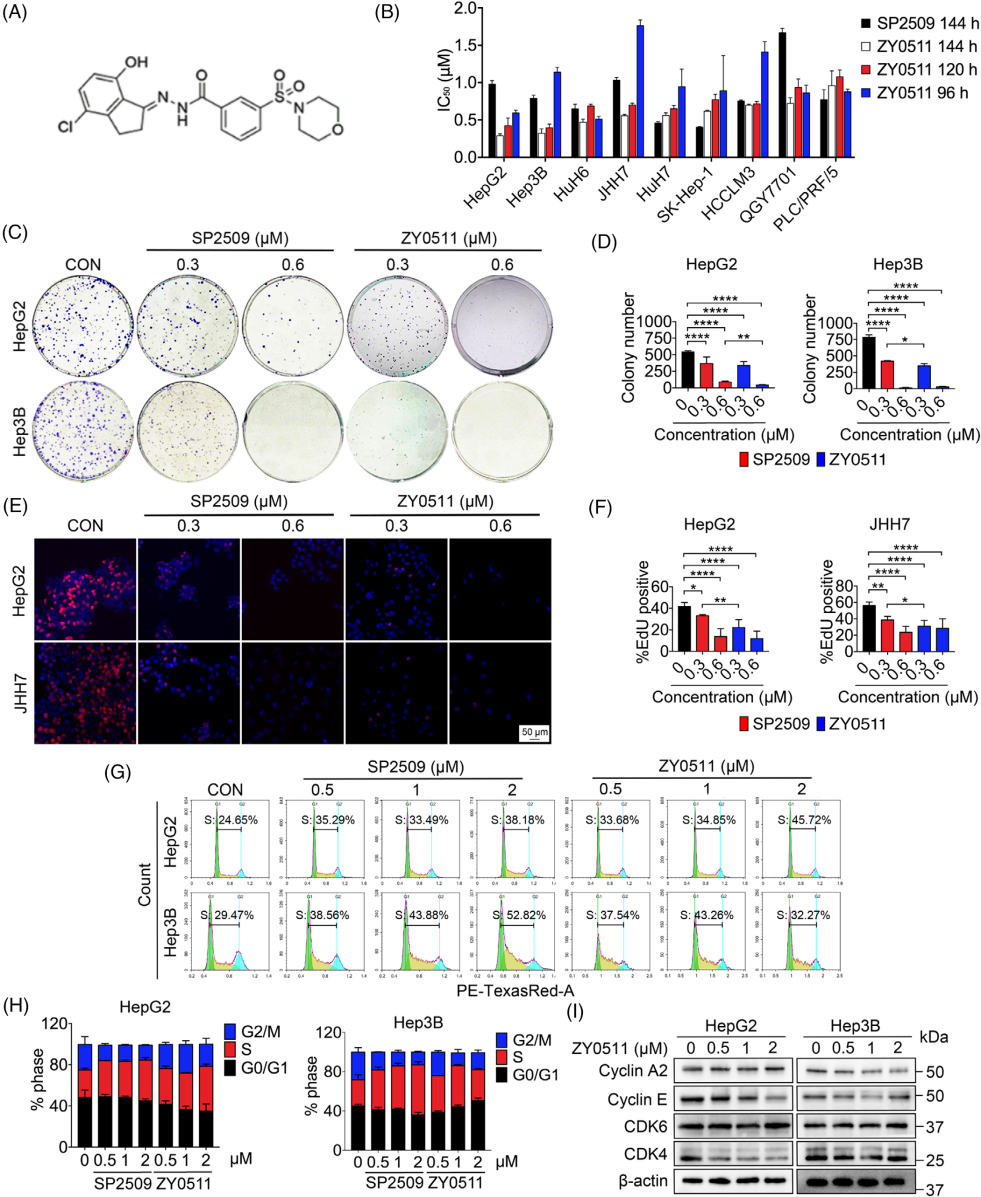
ZY0511 suppresses the proliferation of human hepatocellular carcinoma (HCC) cells in vitro. (A) The molecular structure of ZY0511. (B) The IC_50_ of SP2509 and ZY0511 on nine human HCC cells (*n* = 3). (C‐D) Representative images (C) and quantification (D) of colony formation assay in HCC cells post‐exposure to different concentrations of SP2509 and ZY0511 for 14 days (*n* = 3). (E‐F) Representative images (E) and quantification (F) of EdU incorporation assay in HCC cells post‐exposure to different concentrations of SP2509 and ZY0511 for 96 h (red: EdU; blue: nucleus; *n* = 3). Scale bar, 50 μm. (G‐H) Representative images (G) and quantification (H) of cell cycle in HCC cells post‐exposure to different concentrations of SP2509 and ZY0511 for 24 h (*n* = 3). (I) The protein expression of cyclin‐dependent kinases and cyclins by western blot in HCC cells post‐exposure to different concentrations of ZY0511 for 24 h. β‐actin was the reference protein. Data are presented as mean ± SD. *p < 0.05, **p < 0.01, ****p < 0.0001 versus control.

To investigate the cell proliferative inhibition potential of ZY0511 against human HCC cells, we detected the proliferation of multiple human HCC cell lines exposed to ZY0511 for different periods by CCK‐8 assay. SP2509 was selected as a reference compound as it possesses a similar benzo hydrazide structure to ZY0511. The results showed that ZY0511 strongly inhibited the proliferation of nine HCC cell lines in a time‐ and concentration‐dependent manner, with half‐maximal inhibitory concentration (IC_50_) values ranging from 0.2 to 1.0 μΜ post‐treatment for 144 h, which was about two‐fold stronger than SP2509 (Figure 1A,B). Meanwhile, we compared the efficiency of ZY0511 with other typical LSD1 inhibitors GSK2879552 and ORY1001, which are tranylcypromine‐based compounds and possess trans‐2‐phenylcyclopropylamine structure. The results showed that compared with ZY0511 and SP2509, both GSK2879552 and ORY1001 exhibited much weaker proliferation inhibitory effects against HCC IC_50_ ≥ 28.57 μM; Figure 1B, Table S1). Consequently, colony formation and 5‐Ethynyl‐2'‐deoxyuridine (EdU) incorporation assays were employed for validating the anti‐proliferative effects of ZY0511. We found that ZY0511 efficiently reduced the number and size of colonies in a concentration‐dependent manner (Figures 1C,D and S3A). ZY0511 exposure also significantly decreased the number of EdU‐positive HCC cells, with inhibition rates ranging from 9.2% (PLC/PRF/5) to 99.9% (Hep3B; Figures 1E,F and S3B). Moreover, ZY0511 treatment drove cell cycle S phase stoppage and downregulated the expressions of cyclin‐dependent kinases and cyclins in HCC cells (Figure 1G–I). Compared with SP2509, ZY0511 exhibited stronger efficiency against HCC cells proliferation in the above assays.

Furthermore, we observed that the cell sensitivity against ZY0511 partly correlated with the basal levels of both LSD1 mRNA and protein (Figure S3C,D). For example, HepG2 and JHH7 cells, with high expression of LSD1, were sensitive to ZY0511 treatment; whereas PLC/PRF/5 cell with low expression of LSD1 was less sensitive. Interestingly, Hep3B cell with low expression of LSD1 was sensitive to ZY0511 treatment, which might be due to the deletion of *P53*, or dysregulations in other genes.

Moreover, the migrative properties of HCC cells were also suppressed by ZY0511 treatment (Figure S4A). Together, these results indicated that ZY0511 has significant proliferation inhibitory effects against HCC cells in vitro.

### 2.3 ZY0511 inhibits HCC cells proliferation by specifically interacting with LSD1

Our previous study showed that ZY0511 strongly inhibited LSD1 activity with an IC_50_ value of 1.7 Nm.^23^ To further investigate the specificity of ZY0511 against LSD1, we detected the inhibitory effect of ZY0511 (1 μM) against 104 cancer‐related kinases. ZY0511 at a high concentration of 1 μM only slightly inhibited the activities of several kinases with inhibition rates above 50%, such as ATM, Flt4, ATR/ATRIP, and DNA‐PK, and showed no effect against other kinases, including FGFR, VEGFR, and PDGFR. The IC_50_ values against these selected kinases were as follows: ATM, IC_50_ = 9.80 μM; ATR/ATRIP, IC_50_ = 9.59 μM; Flt4, IC_50_ > 10 μM; DNA‐PK, IC_50_ > 10 μM; IGF‐1R, IC_50_ > 10 μM (Tables S2 and S3, Figure S5). These data suggested that ZY0511 possesses a strong and specific inhibitory effect against LSD1 but no inhibitory effect against cancer‐related kinases.

**FIGURE 2:**
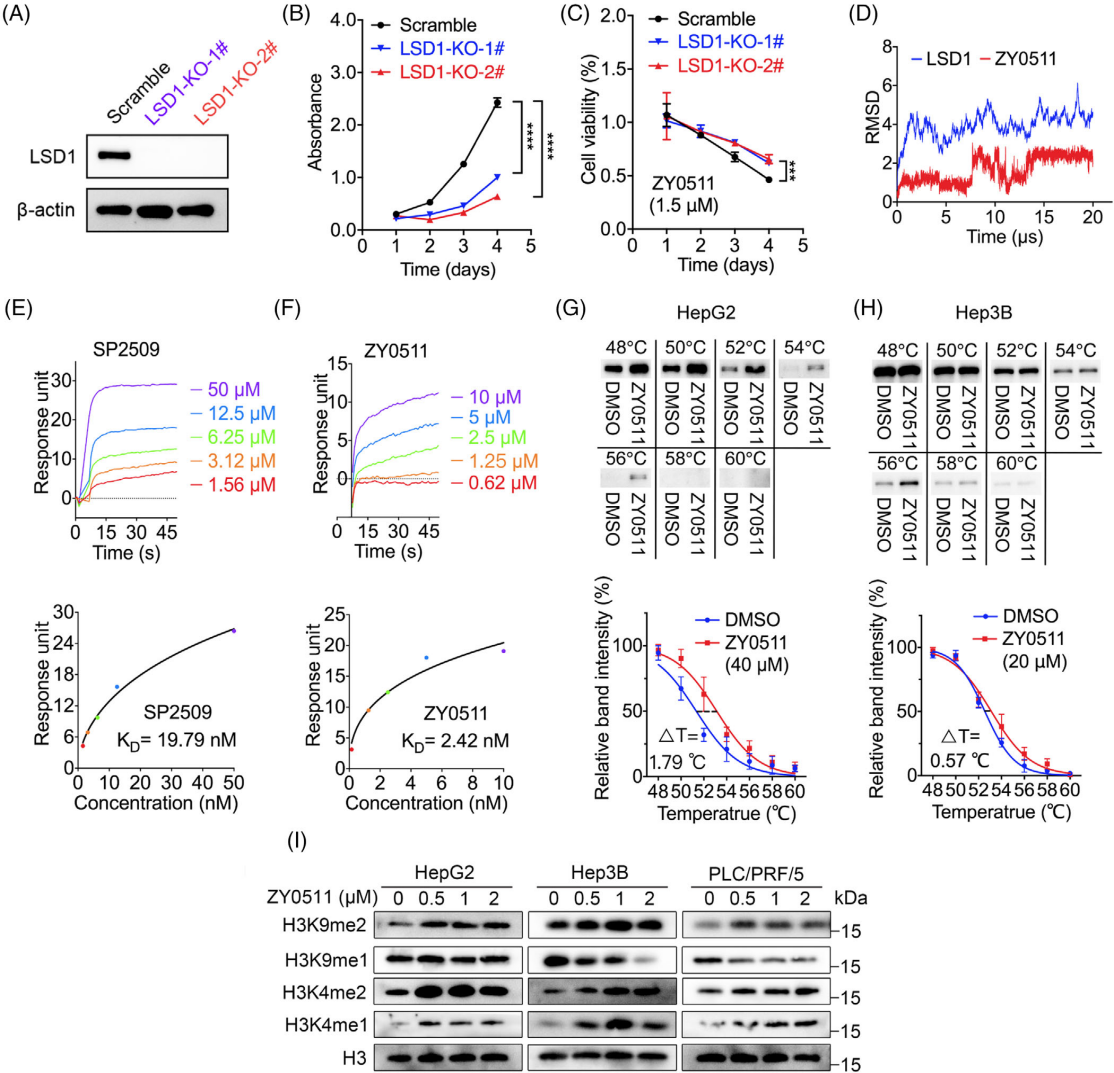
ZY0511 inhibits HCC cells proliferation by specifically interacting with lysine‐specific histone demethylase 1 (LSD1). (A) Immunoblot analysis of LSD1 in JHH7‐scramble and LSD1 knockout clones. (B) The growth curve of JHH7‐scramble and LSD1 knockout clones (*n* = 3). (C) The cell viability of JHH7‐scramble and LSD1 knockout clones post‐ZY0511 treatment (*n* = 3). (D) The root‐mean‐square deviation (RMSD) of ZY0511 in complex with LSD1. (E‐F) The Biacore sensorgrams (above) and saturation curve (below) of SP2509 (E) and ZY0511 (F) binding to LSD1 protein. (G‐H) Representative images (above) and quantification (below) of cellular thermal shift assays melt curve from 48 to 60°C of HepG2 (G) and Hep3B (H) cell lysates with DMSO or ZY0511 incubation (*n* = 3). (I) Immunoblot analysis of H3K4me1, H3K4me2, H3K9me1, and H3K9me2 in HCC cells post‐ZY0511 treatment. All experiments were performed at three independent occasions. Data are presented as mean ± SEM. ***p < 0.001, ****p < 0.0001 versus scramble.

To further confirm that the cell proliferation inhibitory effect of ZY0511 was dependent on LSD1 expression, we constructed LSD1 knockout JHH7 cells via CRISPR‐Cas9 technology. The successful LSD1 knockout was confirmed by western blot analysis (Figure 2A). The cell proliferation assay showed that the proliferation of JHH7 cells with LSD1 knockout was significantly inhibited with inhibitory rates of 58.71%–73.67% (Figure 2B). We then detected the proliferation of LSD1 knockout JHH7 cells after ZY0511 treatment for 96 h. The results showed that LSD1 knockout decreased the sensitivity of JHH7 cells to ZY0511 treatment with a percentage of 20% (Figure 2C), indicating that ZY0511 suppressed HCC cells proliferation in an LSD1‐dependent manner.

Moreover, we performed 20‐μs molecular dynamic simulations on the docking model of ZY0511 and LSD1 complex to further confirm the interaction of ZY0511 with LSD1. Our previous study of the computer‐based molecular docking showed that ZY0511 bound to LSD1 (PDB ID: 5LHH) at glutamine358 (Q358) of AOL domain via hydrogen bond.^21^ Thus, RMSD for LSD1 and ZY0511 were assessed based on initial structural formats. The results showed that the ZY011 treatment decreased the RMSD to 2.2 Å, compared with 4 Å of that in the initial system over the 20‐μs MD simulation (Figure 2D). The low RMSD indicated that ZY0511/LSD1 system was a conforming system without large conformational changes, suggesting that ZY0511 bound with LSD1.

We further investigated the binding affinity between ZY0511 and LSD1 by surface plasmon resonance (SPR) method with a Biacore. SP2509 was selected as a positive control. The result showed that both ZY0511 and SP2509 directly bound to LSD1 protein. ZY0511 bound to LSD1 with calculated dissociation constants (K_D_) of 2.42 nM, which was stronger than that of SP2509 (K_D_ = 19.79 nM; Figure 2E,F).

To detect the bind of ZY0511 to LSD1 protein in HCC cells, we subsequently performed cellular thermal shift assays (CETSA). The results showed that the thermostability of LSD1 raised significantly and thermal shifts increased from 0.57 to 1.79°C post‐ZY0511 treatment, supporting that ZY0511 bound with LSD1 in HCC cells (Figures 2G,H and S4B). Moreover, we found that the higher concentration (40 μM) of ZY0511 was needed to increase the thermostability of LSD1 in HepG2 and JHH7 cells than that (20 μM) in Hep3B and PLC/PRF/5 cells, which was consistent with the high protein level of LSD1 in HepG2 and JHH7 cells than that of Hep3B and PLC/PRF/5 cells.

Since LSD1 demethylates H3K4me1/2 and H3K9me1/2, we detected the methylation modification levels of H3K4 and H3K9 in HCC cells post‐ZY0511 treatment. The results revealed that H3K4me1, H3K4me2, and H3K9me2 were upregulated following ZY0511 treatment (Figure 2I), indicating that ZY0511 inhibited the demethylating function of LSD1. Collectively, the above studies suggested that ZY0511 specifically interacts with LSD1, thus inhibiting HCC cell proliferation.

### 2.4 ZY0511 inhibits HCC growth and is well tolerated in vivo

**FIGURE 3:**
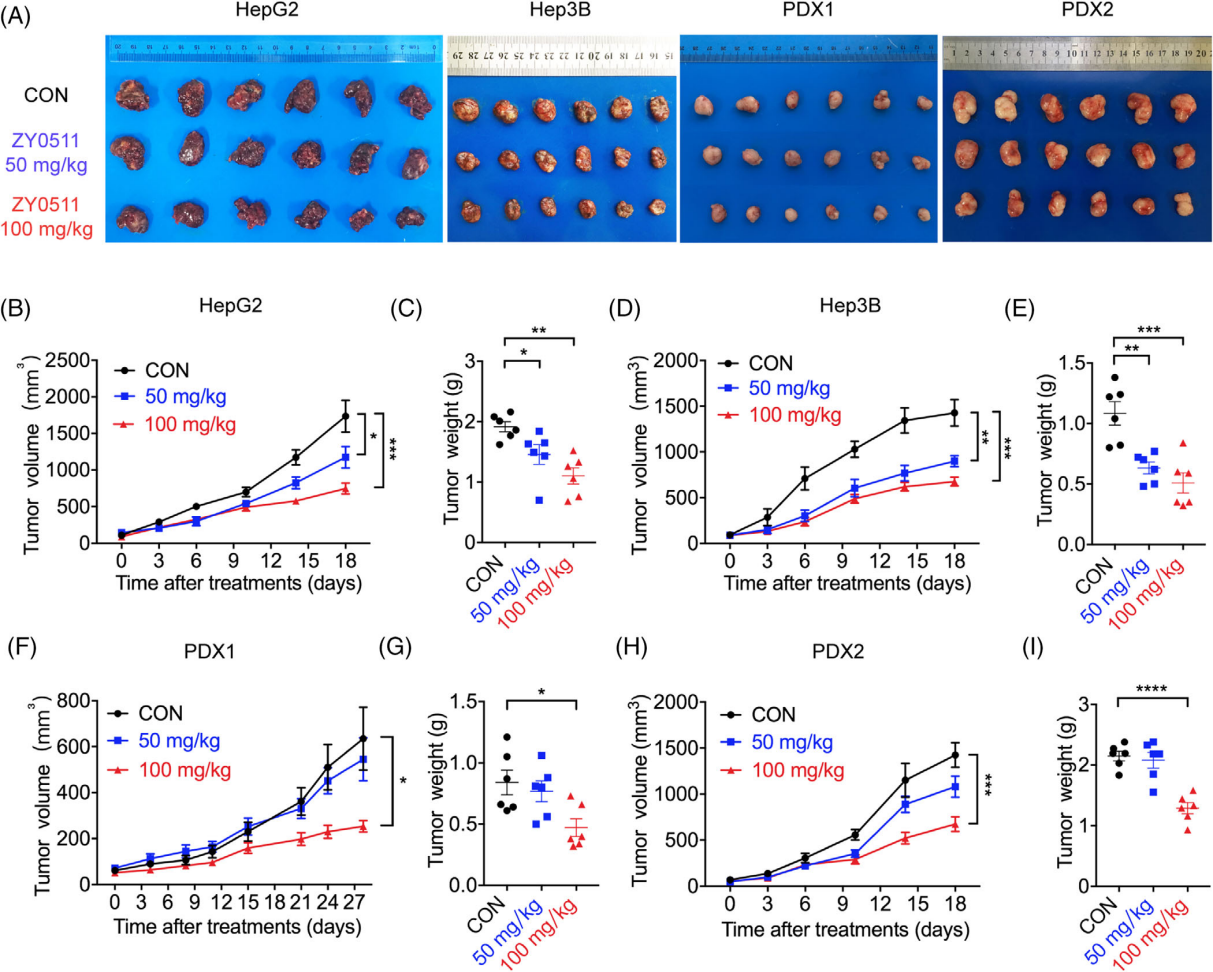
ZY0511 inhibits tumor growth in vivo. (A) Representative images of isolated HepG2, Hep3B, PDX1, and PDX2 tumors from mice at the end of ZY0511 treatment. (B‐C) Growth curve (B) and tumor weight (C) of HepG2 xenograft models post‐ZY0511 treatment. (D‐E) Growth curve (D) and tumor weight (E) of Hep3B xenograft models post‐ZY0511 treatment. (F‐G) Growth curve (F) and tumor weight (G) of PDX1 xenograft models post‐ZY0511 treatment. (H‐I) Growth curve (H) and tumor weight (I) of PDX2 xenograft models post‐ZY0511 treatment. *n* = 6 mice per group. Data are presented as mean ± SEM. *p < 0.05, **p < 0.01, ***p < 0.001, ****p < 0.0001 versus control.

To evaluate whether inhibition of LSD1 by ZY0511 suppresses HCC growth in vivo, two cell‐derived xenograft (CDX) models (HepG2 and Hep3B) and two patient‐derived xenograft (PDX) models were established, and ZY0511 was intraperitoneally (i.p.) administered once tumor volume reached approximately 100 mm^3^. The results showed that ZY0511 treatment significantly suppressed HCC cell growth in vivo (Figure 3A). For HepG2 and Hep3B models, tumor growth inhibition (TGI) reached 58.29% and 52.70%, respectively, after ZY0511 treatment at a dose of 100 mg/kg for 18 days of treatment (*n* = 6; Figure 3B–E). For PDX models, ZY0511 treatment at a dose of 100 mg/kg exhibited TGI values ranging from 39.95% to 62.17% (*n* = 6; Figure 3F–I). The different TGI values in 2 PDX models might be due to the heterogeneity in HCC patients. Moreover, ZY0511 treatment did not change the body weight of mice, compared with that of control (Figure S6A–D). Together, these results demonstrated that ZY0511 has significant inhibitory effects against HCC xenograft tumors in vivo.

At the end of the experiment, the mice were sacrificed, and tumor tissues were removed for pathological examination. Hematoxylin and eosin (H&E) staining of tumor tissues showed irregular cell morphology, remarkable atypia, and deep nuclear staining, suggesting that the tumor xenograft models were successfully established. The expression of Ki67 and proliferating cell nuclear antigen (PCNA) in tumor tissues was markedly decreased by ZY0511 (Figure S6E–H), further demonstrating the proliferation inhibitory potency of ZY0511 in vivo.

Furthermore, acute toxicity assays were performed to detect the toxicity of ZY0511. The result showed that the no observed adverse effect level of ZY0511 is 400 mg/kg. The blood and biochemical analysis showed that ZY0511 administration did not change the blood routine indexes including red blood cell, white blood cell, lymph cell, and so on (Figure S7A), and the important liver and kidney function indexes, such as alanine aminotransferase, aspartate aminotransferase, and creatinine (Figure S7B). No pathological abnormality of the main organs (heart, liver, spleen, lung, and kidney) was observed in the acute toxicity assay (Figure S7C). SP2509 administration exhibited similar results with ZY0511 (Figure S7A–C). Collectively, ZY0511 is well tolerated in vivo.

### 2.5 ZY0511 modulates the expression of LSD1 downstream genes in HCC cells

**FIGURE 4:**
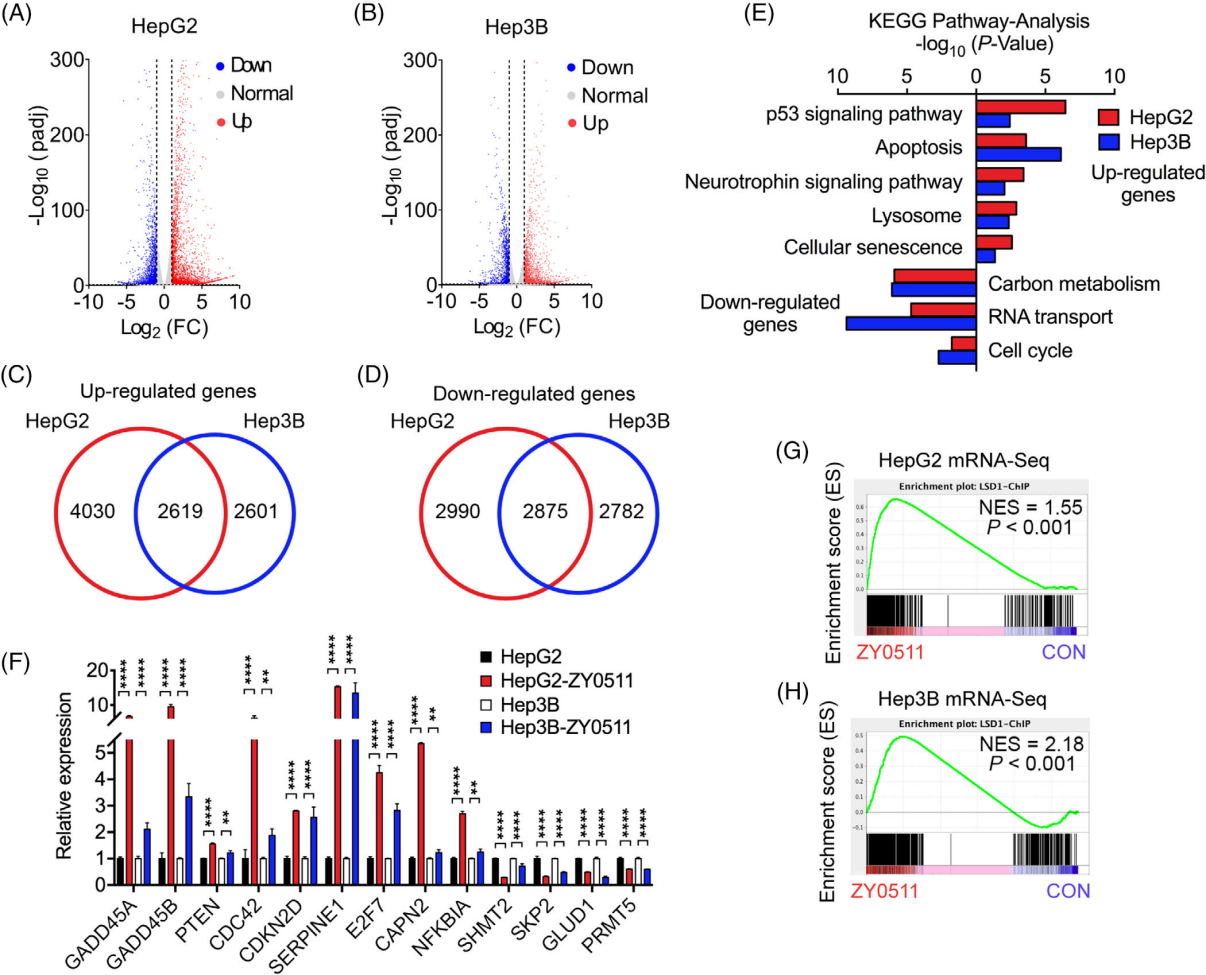
Multivariate gene expression post‐ZY0511 exposure in HCC cells. (A‐B) Volcano plot of mRNA sequencing (mRNA‐seq) results of HepG2 (A) and Hep3B (B) cells post‐ZY0511 exposure (red refers to upregulation, blue refers to downregulation, whereas gray refers to no meaningful change; *n* = 3). (C‐D) Venn diagram referring to overlap of upregulated (C) and downregulated (D) genes between HepG2 and Hep3B cells post‐ZY0511 exposure. (E) Representative Kyoto Encyclopedia of Genes and Genomes (KEGG) pathways analysis of upregulated and downregulated genes post‐ZY0511 treatment. (F) Representative upregulated and downregulated genes post‐ZY0511 exposure (*n* = 3). (G‐H) Gene Set Enrichment Analysis plots showed enrichment of gene sets in HepG2 (G) and Hep3B (H) cells treated with 2 μM ZY0511 for 48 h.

To explore the molecular mechanism by which ZY0511 inhibits the proliferation of HCC cells, mRNA sequencing (mRNA‐seq) technology was applied for assessing the effect of ZY0511 on global genes expression in HepG2 and Hep3B cells. Venn analysis revealed that 2619 upregulated differentially expressed genes (DEGs) and 2875 downregulated DEGs were detected in ZY0511‐treated HepG2 and Hep3B cells (Figure 4A–D). Kyoto Encyclopedia of Genes and Genomes (KEGG) pathway analysis revealed that the upregulated DEGs were mainly involved in p53 signaling pathway, apoptosis, and cellular senescence signaling pathway, while the downregulated DEGs enriched in carbon metabolism, RNA transport, and cell cycle signaling pathway (Figure 4E). Of note, the representative DEGs, including upregulated DEGs, such as *GADD45A* and *GADD45B*, and downregulated DEGs, such as *SHMT2* and *SKP2* (Figure 4F), were the common genes enriched to the above signaling pathways. Furthermore, Gene Set Enrichment Analysis (GSEA) employing LSD1 downstream gene sets from the website (http://cistrome.org/CistromeCancer/) revealed variations in a sub‐group of transcriptional programs within HepG2 and Hep3B cells upon ZY0511 treatment (Figure 4G,H), respectively, suggesting that ZY0511 drove dysregulations in the downstream genes of LSD1.

### 2.6 ZY0511 upregulates GADD45B expression by inhibiting LSD1 and inducing H3K4me2 in HCC cells

**FIGURE 5:**
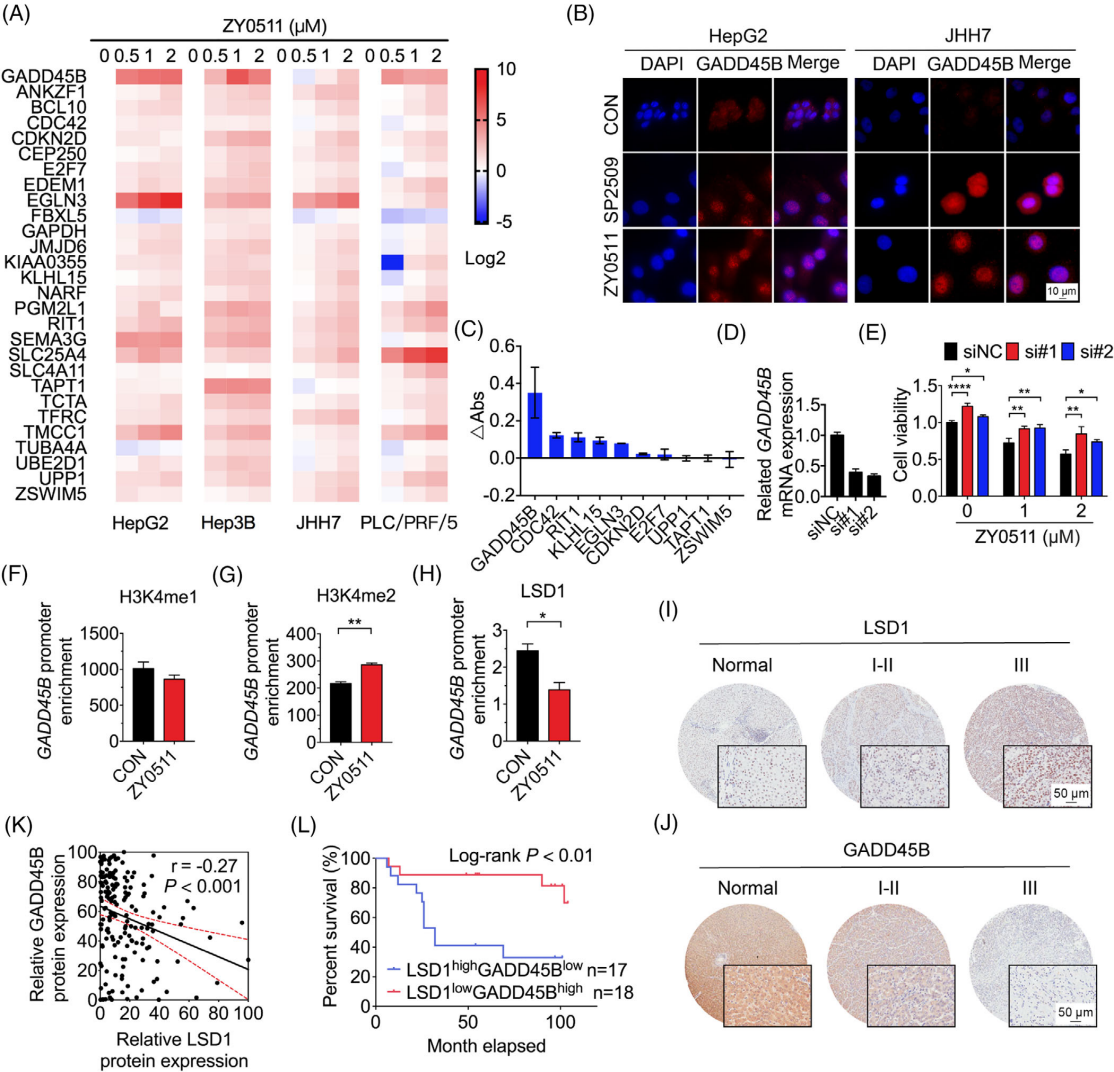
ZY0511 induces GADD45B expression in HCC cells and DNA‐damage‐inducible gene 45beta (GADD45B) is a target of LSD1. (A) Real‐time quantitative polymerase chain reaction (RT‐qPCR) validation of 28 selected upregulated genes in HepG2, Hep3B, JHH7, and PLC/PRF/5 cells. (B) Representative images of immunofluorescence assay using HCC cells post‐SP2509 or ZY0511 for 48 h (red: GADD45B; blue: nucleus). Scale bar, 10 μm. (C) HepG2 cells were transfected with individual siRNAs targeting 10 selected upregulated genes, and absorbance change was detected by using MTT assay (*n* = 3). (D) mRNA expression levels of *GADD45B* transfected with siGADD45B (#1, #2) and siNC. (E) The proliferation of HCC cells post‐ZY0511 (0.3 μM) treatment with interference of *GADD45B* expression for 96 h (*n* = 3). (F‐H) The enrichment of H3K4me1 (F), H3K4me2 (G) and LSD1 (H) in GADD45B promoter of HepG2 cells by chromatin immunoprecipitation (ChIP)‐qPCR post‐ZY0511 (2 μM) treatment for 48 h (*n* = 3). (I‐J) Representative immunohistochemistry images of LSD1 (I) and GADD45B (J) in HCC tissues with different stages in tissue chip. Scale bar, 50 μm. (K) Correlation between the protein expression levels of GADD45B and LSD1 in HCC tissues. (L) Kaplan–Meier survival curves for LSD1 and GADD45B expression in HCC patients. Data are presented as mean ± SD. *p < 0.05, **p < 0.01, ****p < 0.0001 versus vehicle.

Since ZY0511 significantly increased the levels of H3K4me1/2, which could promote genes expression, we focused on the correlation between the upregulated DEGs and ZY0511 in HCC. A total of 28 potential target genes of LSD1 were selected (Table S4). The commonality of these selected genes was: (1) the expression of these genes detected by mRNA‐Seq was upregulated significantly post‐ZY0511 treatment; (2) the expression of these genes was strongly correlated with *KDM1A* expression in TCGA database; (3) in OncoLnc survival analysis website, the absolute values of Cox were large, suggesting that these genes were important for HCC. By using real‐time quantitative polymerase chain reaction (RT‐qPCR) assay, we validated the upregulation of 28 candidate genes in HCC cells, including *CDC42*, *CDKN2D*, and *GADD45B*, which were consistent with the mRNA‐seq results (Figure 5A). Notably, we found that mRNA expression of *GADD45B* prominently increased in HepG2 cells after ZY0511 treatment (Figure 5A). Immunofluorescence assay confirmed that the protein expression of GADD45B was markedly upregulated in HCC cells (Figure 5B). Consequently, we selected 10 dominantly dysregulated genes and detected the proliferation of HepG2 cells after silencing these genes by using small interfering RNA (siRNA) library screening. Importantly, GADD45B knockdown not only resulted in the highest proliferation increase in HCC cells (Figure 5C) but also reversed ZY0511‐induced proliferation inhibition of HepG2 cells (Figure 5D,E), suggesting that GADD45B is an important downstream molecule of LSD1 and plays a critical role in ZY0511 efficiency.

To further investigate whether GADD45B expression induced by ZY0511 was regulated by LSD1, chromatin immunoprecipitation (ChIP) qPCR assays were performed. The results showed that LSD1 linked to the promoter of GADD45B and ZY0511 remarkably decreased LSD1 enrichment and increased H3K4me2 enrichment in GADD45B gene promoter (Figure 5F–H). Moreover, the protein expression of LSD1, H3K4me1/2, and GADD45B were investigated by immunohistochemistry (IHC) staining in the subcutaneous tumors post‐ZY0511 administration in vivo. ZY0511 upregulated the expression of H3K4me1/2 and GADD45B instead of LSD1 (Figure S8A–D), which was consistent with the trend in vitro (Figures 2I and 5B). Collectively, these data indicated that GADD45B is a direct downstream target of LSD1, and ZY0511 epigenetically upregulates GADD45B expression by inducing H3K4me2 enrichment in GADD45B gene promoter.

### 2.7 The LSD1 level inversely correlates with GADD45B level in human HCC tissues and with the survival rates of patients

To gain insight into all correlations and clinical valorization of GADD45B/LSD1 in HCC, we investigated the mRNA levels of *GADD45B* and *KDM1A* genes in HCC cells by using Cancer Cell Line Encyclopedia dataset. The results showed that the mRNA level of *GADD45B*, instead of *GADD45A* and *GADD45G*, strongly correlated with *KDM1A* in HCC cells (Figure S9A), which is consistent with data from the TCGA (Figure S9B), International Cancer Genome Consortium (Figure S9C), and Gene Expression Omnibus (GEO; Figure S9D) databases. Moreover, RT‐qPCR assay confirmed that the mRNA level of the *GADD45B* gene was inversely correlated with the *KDM1A* level in HCC cells (Figure S9E).

IHC staining was used to validate the protein levels of GADD45B and LSD1 in HCC tissue microarray containing 84 human HCC tissues/NATs. Staining outcome datasets highlighted LSD1 expression was upregulated in HCC tissues, compared with that in NATs (Figures 5I and S9F–I), while GADD45B level was decreased in HCC tissues, compared with that in NATs (Figures 5J and S9J–M). Regarding LSD1, only 15 cases (17.86%) exhibited no or weak immunopositivity, 32 cases (38.10%) exhibited moderate immunopositivity, and 37 cases (44.05%) exhibited strong immunopositivity in the tumor tissues (Figure S9F), suggesting LSD1 expression in the HCC tissues was predominantly upregulated in comparison with NATs (Figure S9G). Regarding IHC data for GADD45B, 54 cases (64.28%) exhibited no or weak immunopositivity, 21 cases (25.00%) exhibited moderate immunopositivity, while 9 cases (10.71%) exhibited strong immunopositivity in the HCC tissues (Figure S9J). Collectively, the expression of GADD45B in HCC tissues was predominantly lower than that in NATs (Figure S9K). By using IHC datasets, we continued to analyze the putative associations of GADD45B with LSD1. The results showed that HCC tissues with upregulated LSD1 tended to exhibit a downregulated GADD45B expression, while the protein expression of GADD45B was inversely associated with that of LSD1 in HCC tissues (*r* = −0.27, *p* < 0.001; Figure 5K). We further analyzed the correlation of GADD45B and LSD1 expression levels with HCC progression and found LSD1 level in HCC tissues was positively linked to the advanced stage (Figure S9H) and poor survival rate in HCC patients (Figure S9I), which is in line with data from TCGA (Figures S1D and S2A). In contrast, GADD45B expression level in HCC tissues was inversely associated with advanced stage (Figure S9L) and poor survival rate of HCC patients (Figure S9M). These results were consistent with the data from TCGA (Figure S9N–P). Notably, the patients with upregulated LSD1 and downregulated GADD45B exhibited worse survival rates than the patients with downregulated LSD1 and upregulated GADD45B (Figure 5L). In summary, these data indicated that LSD1 level inversely correlates with GADD45B level in HCC tissues and correlates with poor survival rates of HCC patients.

### 2.8 The combination of ZY0511 and DTP3 synergistically inhibits the proliferation and growth of HCC cells

**FIGURE 6:**
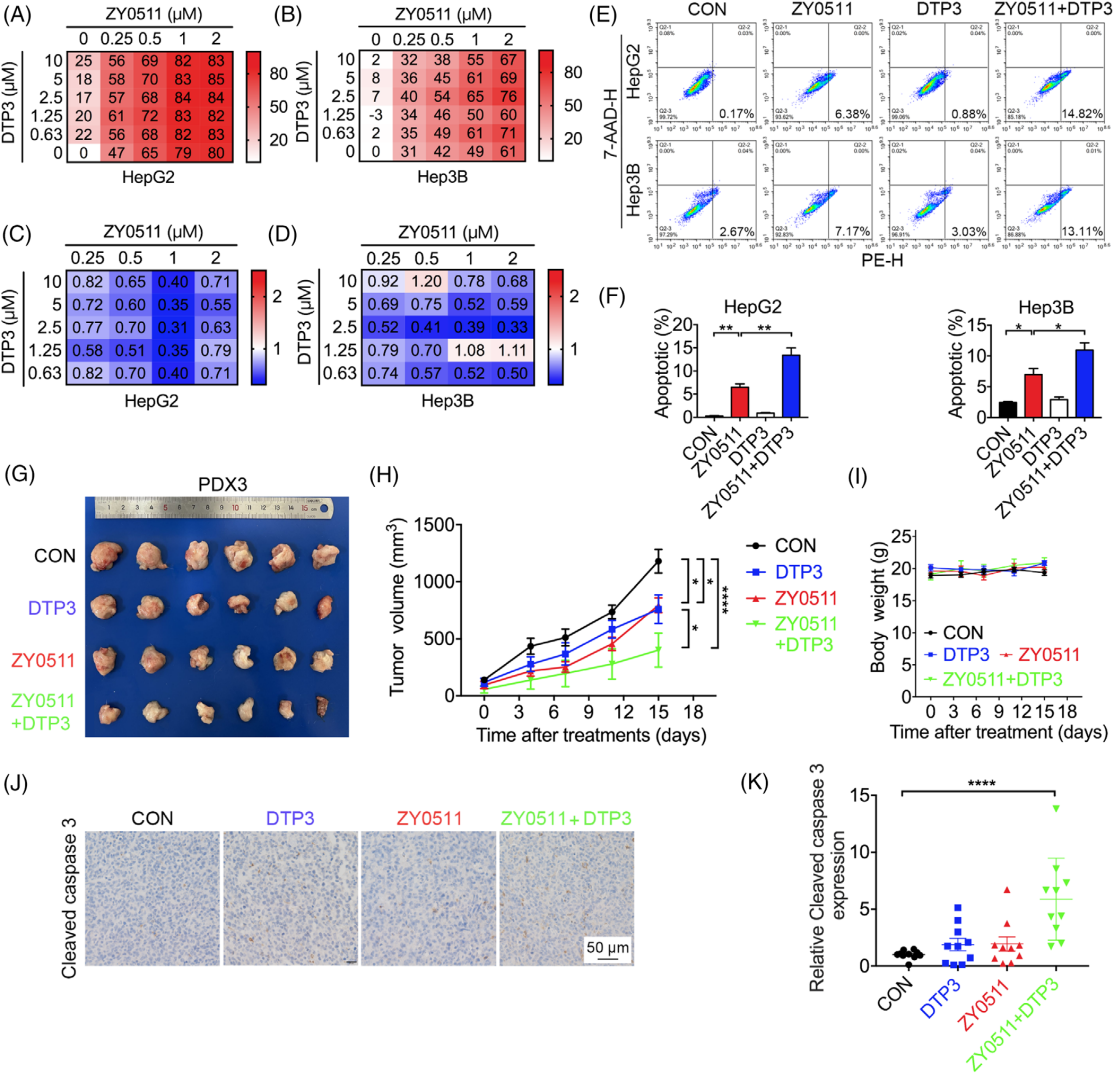
Combination of ZY0511 and DTP3 synergistically inhibit HCC cells proliferation in vitro and in vivo. (A‐B) Inhibition rate of HepG2 (A) and Hep3B (B) cells determined by MTT assay after treatment of ZY0511 and DTP3 at the indicated concentration for 96 h. (C‐D) CI scores of HepG2 (C) and Hep3B (D) cells by CompuSyn software post‐ZY0511 and DTP3 treatment at the indicated concentration for 96 h. (E‐F) Representative images (E) and quantification (F) of apoptosis assay in HepG2 and Hep3B cells post‐ZY0511 in combination with DTP3 treatment. (G‐H) Representative image (G) and growth curve (H) of isolated PDX3 tumors post‐ZY0511 or in combination with DTP3 treatment for 15 days. (I) Body weight curve of mice with PDX3 subcutaneous tumors post‐ZY0511 or in combination with DTP3 treatment for 15 days. *n* = 6 mice per group. (J‐K) Representative immunohistochemistry images (J) and quantification (K) of Cleaved caspase 3 in PDX3 tumors after ZY0511 treatment combined with DTP3 administration at the end of the experiment. Scale bar, 50 μm. Data are presented as mean ± SEM. *p < 0.05, **p < 0.01, ****p < 0.0001 versus vehicle.

GADD45B is a multifunctional protein that inhibits cell proliferation and induces cell cycle arrest and apoptosis by interplaying with cellular proteins. However, its pro‐apoptotic effect is weakened when it binds and abrogates the catalytic activity of mitogen‐activated protein kinase (MAPK) kinase 7 (MKK7) and in turn suppresses the activation of the pro‐apoptotic c‐Jun N‐terminal kinase (JNK) signaling pathway. DTP3, a small molecular peptide, specifically binds to MKK7 and thus enhances the pro‐apoptotic effect of GADD45B but does not affect the anti‐proliferation ability of GADD45B.^24^ We inferred that a combination of DTP3 with ZY0511 might potentiate the proliferation inhibitory effect of ZY0511. As expected, a combination of ZY0511 with DTP3 synergistically suppressed the proliferation of HCC cells, combination index (CI) < 0.75, at almost all analyzed concentrations (Figure 6A–D). Compared with ZY0511 alone, ZY0511 in combination with DTP3 significantly increased HCC apoptotic rates from 6.49% to 13.40% in the HepG2 cells and 6.98% to 10.96% in the Hep3B cells (Figure 6E,F).

Then, we investigated the anti‐tumor activities of a combination of ZY0511 with DTP3 in the third PDX model. Compared with the control group, ZY0511 (50 mg/kg) or DTP3 (30 mg/kg) alone inhibited tumor growth with TGI of 32.79% and 35.75%, respectively. When ZY0511 was combined with DTP3, the TGI increased to 66.12%, which exhibited a stronger effect than ZY0511 or DTP3 alone (Figure 6G,H), and no change of mice body weight was observed after ZY0511 and/or DTP3 treatment (Figure 6I). The apoptosis biomarker cleaved caspase‐3 in tumor tissues was upregulated by ZY0511/DTP3 co‐treatment (Figure 6J,K), indicating that this combination enhanced anti‐HCC efficiency by inducing apoptosis.

## 3 DISCUSSION

Despite early diagnosis and interventions improving the cure rate, most patients develop into advanced‐stage HCC, where very limited systemic drugs are available, such as sorafenib, lenvatinib, and regorafenib.^2^, ^3^ Therefore, novel approaches for advanced HCC, especially targeted therapy, are gravely required. To the best of our knowledge, only few studies used LSD1 inhibitors for HCC treatment. The present investigation confirmed LSD1 as a potential target for HCC therapy. Targeting LSD1 by ZY0511, a potent LSD1 inhibitor, effectively suppressed HCC growth in vitro and in vivo by upregulating the expression of GADD45B, a direct target gene of LSD1. High‐level LSD1 and low‐level GADD45B were linked to poor survival rates in HCC patients. Combinatory use of ZY0511 and DTP3, which specifically enhances the pro‐apoptotic effects of GADD45B, synergically inhibited the growth of HCC. These findings highlight therapeutic perspectives of LSD1 inhibitor‐based novel combinatorial strategy for treating HCC.

Small molecular drugs targeting epigenetic facets attracted rising attention in liver cancer treatment, including HDAC inhibitors Belinostat and Panobinostat and DNMT inhibitor azacytidine.^25^, ^26^, ^27^, ^28^, ^29^ Over the last decade, although LSD1 has become a candidate therapeutic target for drug development against various malignancies, no FDA‐approved LSD1 inhibitor has been used for HCC therapy.^30^ Our results from TCGA databases suggested that both mRNA and protein levels of LSD1 were highly evaluated in human HCC tissues and correlated with the poor survival rates of patients, and LSD1 knockout significantly inhibited the proliferation of HCC cells. These results are consistent with the previous report that inhibition of LSD1 by siRNA inhibited the growth of HepG2 subcutaneous tumor,^12^ supporting that LSD1 plays an important role in HCC progression and its potential value as a promising target of HCC therapy. ZY0511 is a novel and effective LSD1 inhibitor developed by our research group based on computer‐aided drug design and high‐throughput screening.^23^ Our present work demonstrated that ZY0511 possessed a strong anti‐HCC efficiency both in vitro and in vivo, exhibiting an anti‐HCC efficiency superior to other LSD1 inhibitors, such as GSK2879552 and ORY1001. GSK2879552 and ORY1001 derives from trans‐2‐phenylcyclopropylamine hydrochloride,^31^, ^32^ whereas ZY0511 and SP2509, similar to ZY0511 in structure, possesses the benzohydrazide structure.^21^, ^33^ As a result, these two kinds of inhibitors exhibit varying inhibitory effects against tumor cells. Besides structure difference, it is reported that these LSD1 inhibitors interacted with LSD1 by different binding sites.^34^, ^35^, ^36^ GSK2879552, an irreversible inhibitor that covalently bound to FAD of LSD1,^32^ whereas ZY0511 may be allosteric inhibitor that interacted with the allosteric site of LSD1 with the neighboring amino acids (Gly358, Cys360, Leu362, Asp375, and Glu379)^21^, ^37^ although the binding site of ZY0511 with LSD1 need to be experimentally confirmed by cocrystallization. The allosteric inhibitors were well tolerated in vivo, compared with irreversible inhibitors. Thus, the difference in structure and binding site with LSD1 may be responsible for the effect difference of LSD1 inhibitors, and allosteric inhibitors exerted broad antitumor effects. Moreover, we compared the antitumor effects of ZY0511 with SP2509. It is reported that SP2509 blocked the proliferation of Ewing sarcoma, retinoblastoma, and HCC.^15^, ^38^, ^39^ Our study demonstrated that ZY0511 exhibited stronger efficiency than SP2509 not only in the IC_50_ value and K_D_ against LSD1 but also in IC_50_ value against some HCC cells in vitro. Collectively, our results suggested that ZY0511 exhibited a stronger anti‐HCC effect than SP2509.

Elucidation of the mechanisms by which LSD1 inhibitor suppresses HCC progression is the basis for its application for HCC treatment. Few studies have investigated the possible mechanisms underlying the action of LSD1 in HCC proliferation,^12^ metastasis,^34^ and drug resistance.^9^ Sakamoto et al. reported that inhibition of LSD1 by siRNA restored H3K4 methylation, activated mitochondrial respiration, reduced glucose uptake, and inhibited glycolytic activity, thus inhibiting the growth of HepG2 subcutaneous tumor.^12^ LSD1 also induced stemness and chemotherapy tolerance in the liver Lgr5^+^ hepatoma‐initiating cells through LSD1/pricle1/adenomatous polyposis coli (APC)/β‐catenin signal transduction axis, leading to the drug resistance exhibited in HCC.^9^ In addition, LSD1 expression was induced by Notch signaling through deacetylation, which activated the self‐renewal of HCC stem cells in tandem with LSD1 expression in cancer‐related fibroblasts, thus promoting the progression of HCC.^11^ In our study, ZY0511 induced GADD45B expression by increasing H3K4me1/2 enrichment in the promoter of GADD45B gene in HCC cells, revealing a novel downstream target gene of LSD1.

Moreover, the cellular function of GADD45B is dependent on the types of stimuli, cell types, and the interaction of its partner proteins or signal pathways.^18^, ^24^, ^40^ GADD45B is found to be an independent risk factor for CRC,^18^ epithelial ovarian cancer,^40^ and multiple myeloma.^24^ In HCC, the promoter region of GADD45B is hypermethylated, which is associated with low or loss of expression. Treatment with DNMT inhibitor 5‐azacytidine induces re‐expression of GADD45B and inhibits the proliferation of hepatoma cells.^41^ Thus, GADD45B may be an advantageous factor for suppressing HCC. Our investigation confirmed that low GADD45B levels in human HCC tissues were associated with advanced stage and poor patient survival rates, and ZY0511 inhibited HCC cell growth by restoring GADD45B expression in HCC cells. Our findings further supported that GADD45B is a tumor suppressor gene in HCC, which is consistent with the above and previous studies.^42^ The mechanisms underlying GADD45B expression include tumor necrosis factor‐alfa (TNFα) / nuclear factor kappa‐B (NF‐κB), transforming growth factor‐beta (TGFβ) / drosophila mothers against decapentaplegic protein (SMAD) signaling pathway, and DNA methylation,^43^, ^44^ but the role of histone modification on GADD45B regulation is unclear. We revealed a novel mechanism for epigenetic regulation of GADD45B, namely, LSD1 directly downregulated GADD45B expression through demethylating of H3K4me2. However, GADD45B may be only partially responsible for the effect of ZY0511 as LSD1 could regulate genes expression by demethylating H3K4 or H3K9, or non‐histone substrates such as P53. For example, we found that ZY0511 treatment induced the expression of *CDC42*, *CDKN2D, E2F7*, and so forth. The effect of LSD1 on cell cycle or expression of cyclin and CDK were observed in previous studies.^21^, ^22^ Thus, the action mechanism of ZY0511 against HCC need to be further investigated.

Regarding the heterogeneity of HCC, it is difficult to achieve outstanding tumor inhibition through the use of a single drug. LSD1 knockdown or inhibitors, SP2509, and tranylcypromine enhanced the anti‐proliferation or apoptotic effects of regorafenib against HCC cells.^16^ Other LSD1 inhibitors, GSK2879552, and pargyline suppress stem‐like properties of sorafenib‐resistant HCC cells. The combination of SP2509 with sorafenib has an obvious effect on sorafenib‐resistant hepatoma cells,^17^ suggesting that LSD1 inhibitors in combination with other drugs can be a promising strategy for HCC treatment. DTP3, a D‐tripeptide, is the only modulator targeting GADD45B without obvious toxicity to normal tissues. DTP3 blocks the formation of GADD45B/MKK7 complex by binding with MKK7, thus enhancing apoptosis induction.^24^ Our results demonstrated that ZY0511, in combination with DTP3, synergistically inhibited HCC growth. This is the first report of an LSD1 inhibitor in combination with a GADD45B modulator for HCC treatment. In our study, we also evaluated the combination efficiency of ZY0511 with first‐line multikinase inhibitors against HCC cells and only the ZY0511/lenvatinib combination exhibited a degree of combinational effect.

There are some limitations of our study. For example, we cannot obtain the cocrystallization results of ZY0511 with LSD1. Thus, the binding site of ZY0511 with LSD1 cannot be experimentally confirmed. Besides GADD45B, there may be other mechanisms involved in the action of ZY0511, which needs further study. Moreover, the druggability of ZY0511 need to be improved.

In summary, LSD1 inhibitor ZY0511 inhibits the growth of HCC cells both in vitro and in vivo. Mechanistically, it upregulates GADD45B expression through inhibiting LSD1 and subsequently restoring the level of methylated H3K4. In combination of ZY0511 and DTP3 synergistically inhibits HCC growth. Our findings provide a new strategy for HCC targeted therapy and suggest that a combination of ZY0511 and GADD45B modulator can be a promising strategy for HCC therapy.

## 4 MATERIALS AND METHODS

### 4.1 Bioinformatics analysis

To explore the clinical significance and correlation of LSD1 in human HCC tissues, we analyzed the role of *KDM1A* (the gene encodes LSD1 protein) in LIHC and other five common digestive tract tumors, including COAD, READ, PAAD, ESCA and STAD with two TCGA visualization websites UALCAN (http://ualcan.path.uab.edu) and OncoLnc (http://www.oncolnc.org). Moreover, the GEO database GSE78737 was downloaded to analyze the correlation of genes.

### 4.2 Compounds

ZY0511 ((E)‐N'‐(2, 3‐dihydro‐1H‐inden‐1‐ylidene) benzohydrazides), with purity over 99%, was developed at State Key Laboratory of Biotherapy, Sichuan University as described previously.^23^ Figure 1A showed its structural formula. For in vitro experiments, ZY0511 and SP2509 (Selleckchem) were dissolved in dimethyl sulfoxide (DMSO) to a final concentration of no more than 0.1% (v/v). For in vivo assays, ZY0511 was suspended in 3% PEG 4000 and 1.2% Tween‐20, followed by high‐pressure homogenization to form a nanocrystalline suspension. LSD1 inhibitors GSK2879552 (Selleckchem) and ORY1001 (Selleckchem) and GADD45B modulator DTP3 (Selleckchem) was dissolved in sterile water.

### 4.3 Cell lines and cell culture

Human HCC cell lines HepG2, Hep3B, JHH7, HuH7, Sk‐Hep‐1, and PLC/PRF/5 were obtained from American Type Culture Collection (ATCC). Human HCC cell lines HCCLM3 and QGY7701 were obtained from the cell bank of the Chinese Academy of Sciences. RPMI‐1640 or DMEM media (Gibco) supplemented with 10% fetal bovine serum (Gibco) was used to maintain cell culture at 37°C with 5% CO_2_.

### 4.4 Animals

Female mice (Balb/c) and nude mice (Balb/c and NOD/SCID; 6–8 weeks old) were procured from HFK Biotechnology Company with Animal Quarantine Conformity Certificates. Mice were maintained at 21°C and 55% humidity, with a 12 h light/dark cycle and ad libitum food/water.

### 4.5 Patient samples and human HCC tissue specimens

HCC tissue samples for building PDX model were obtained from West China Hospital, Sichuan University. A tissue chip containing 84‐pairs of human HCC tissues and NATs was procured through National Engineering Center for Biochips and was examined histologically with LSD1 (CST, #2139) and GADD45B (Abcam, ab105060) antibodies. Detailed clinicopathologic characteristics of HCC patients are listed in Table S5.

### 4.6 Molecular dynamics (MD

The g_rmsd programs within GROMACS package (version 4.6.7) were used to perform root‐mean‐square deviation (RMSD)^45^. G_energy program of GROMACS was used to calculate the potential, kinetic and total energies. LIGPLOT+ software (version 1.4.5) was employed for analyzing hydrogen bonding/hydrophobic interplays among key residues of LSD1 and ZY0511.^46^

### 4.7 The activity of kinases detection

The activity of ZY0511 against 104 cancer‐related kinases was carried out by Eurofins Pharma Discovery Services (Celle Lévescault, France).

### 4.8 CETSA assay

HCC cells were exposed to ZY0511 (20 or 40 μM) for 60 min under conventional culture conditions. The cells were digested and collected into BD tubes. The supernatant was discarded after centrifugation at 300 ×g for 3 min and re‐suspended in PBS (containing protease inhibitor cocktail), followed by aliquoting into PCR tubes (3 × 10^6^ cells/tube). Samples were heated according to temperature gradient (48, 50, 52, 54, 56, 58, and 60°C) for 3 min and chilled at 4°C for 3 min by PCR thermal block instrument. Samples were exposed to liquid nitrogen freeze/room‐temperature thaw cycles thrice. The supernatant protein from each sample was extracted after centrifugation at 20,000 *g* for 20 min. Protein samples were treated with loading buffer at 4:1, vortexed, and denatured in boiling water for 5 min. Finally, LSD1 expression in protein samples was detected by western blot and quantified by Image J software. CETSA curves were graphed using GraphPad Prism 8 (GraphPad).

### 4.9 LSD1 protein preparation and SPR assay

Truncated human LSD1 (residues 172–836) was purified as described. Briefly, the truncated LSD1 was cloned into a pET28a plasmid and expressed in E*scherichia coli* BL21(DE3) at 18°C (Novagen). The fusion protein was purified by the nickel charged nitrilotriacetic acid (Ni‐NTA, GE Healthcare) with buffer A (20 mM Tris‐HCl, pH 8.5, 10 mM imidazole, 500 mM NaCl, and 2 mM dithiothreitol DTT) and buffer B (20 mM Tris‐HCl, Ph 8.5, 500 mM imidazole, 500 mM NaCl, and 2 mM DTT). Then, LSD1 was digested overnight with Thrombin Protease (Sigma Aldrich) to remove His‐tag and dialyzed against buffer C (20 mM Tris‐HCl, pH 8.5, 150 mM NaCl, and 2mM DTT) overnight at 4°C. The next day, the target protein was applied to Ni‐NTA again to remove the uncleaved His‐tag protein. LSD1 without His‐tag was further purified by an S200 16/600 column (GE Healthcare) in buffer C. The quality of purified LSD1 was checked by sodium dodecyl sulphate‐polyacrylamide gel electrophoresis (SDS‐PAGE).^47^

The binding affinity between LSD1 and ZY0511 or SP2509 was measured using a Biacore biosensor (Biacore AB). LSD1 protein was immobilized on a CM5 sensor chip by the amine‐coupling method. After CM5 chip surface was activated for 10 min using 200 mM 1‐ethyl‐3‐(3‐dimethylaminopropyl) carbodiimide hydrochloride(EDC)/50 mM N‐hydroxysulfosuccinimide (NHS), a total of 2 μL of LSD1 (600 μg/mL) in 10 mM sodium acetate (pH 5.0) was coupled via injection for 15 min at 10 μL/min, followed by injection of 1 M ethanolamine (PH 8.0) to block the remaining activated groups (or deactivate residual amines). To measure kinetics at 25°C, various concentrations of ZY0511 (0.62–10 μM) and SP2509 (1.56–50 μM) were prepared by two‐fold serial dilution in PBSP (PBS pH 7.4, 0.01% P20), followed by flowing over LSD1 chip surface. The binding kinetics was analyzed using BIAevaluation software (version 4.1, Biacore).

### 4.10 Cell proliferation assay

Cell viability was determined using Cell Counting Kit‐8 (CCK8; Dojindo) or 3‐(4, 5‐dimethylthiazol‐2‐yl)‐2,5‐diphenyltetrazoliumbromide (MTT, Sigma Aldrich) assay. A selection of HCC cells was exposed to gradient concentration of ZY0511 for 96–144 h, or exposed to other LSD1 inhibitors including SP2509, GSK2879552, and ORY1001 for 144 h, or exposed to DTP3, which enhanced the apoptosis induction function of GADD45B for 96 h in combination therapy. Consequently, cell viability was determined using a microplate reader (Thermo Fisher) at 450 nm for CCK8 assay, or 570 nm for MTT assay, at different time points. The IC_50_ values and CI values were analyzed and calculated using GraphPad Prism 8 and CompuSyn (version 1.0; http://www.combosyn.com), respectively.

### 4.11 Colony formation assay

Cells were placed into six‐well plates (800–1000 cells/well) and cultured for 24 h, followed by different concentrations (0.3 μM, 0.6 μM) of ZY0511 or SP2509 treatment. After incubation for 10–14 days, cells were fixed with methanol and stained with crystal violet. Clones were imaged, and the numbers of cell clones were counted by using Image J software.

### 4.12 EdU incorporation assay

EdU assay was utilized to detect proliferating cells. HCC cell aliquots were placed within 96‐well plates (4–5 × 10^3^ cells/well) and exposed to different concentrations (0.3 μM, 0.6 μM) of ZY0511 or SP2509 treatment for 96 h. Consequently, cells were stained with Cell‐Light EdU Apollo 488 In Vitro Imaging Kit (Ribobio). EdU‐positive cells were detected by employing high‐throughput screening (HTS; Thermo Fisher), and the EdU incorporation rate was calculated.

### 4.13 Cell‐cycle analysis and apoptosis analysis

For cell‐cycle analysis, HCC cells were seeded in six‐well plates (2–5 × 10^5^ cells/well) and cultured for 24 h, followed by different concentrations (0.5 μM, 1 μM, and 2 μM) of ZY0511 or SP2509 treatment for 24 h. Cells were fixed using 70% ethanol and stained with a Cell Cycle Detection Kit (KeyGEN). For apoptosis analysis, cells were exposed to ZY0511 alone or in combination with DTP3 for 48 h. Cells were harvested and stained with PE Annexin V Apoptosis Detection Kit (BD). The cell cycle or apoptosis induction was detected by using a flow cytometer (Agilent NovoCyte). Results were assessed by NovoExpress software (Agilent NovoCyte).

### 4.14 Single‐cell clone selection

To obtain complete LSD1 knockout clones using the CRISPR‐Cas9 system, HCC cells were transfected with lentiviral for CRISPR targeting of the LSD1 gene. The sgRNA target sequences for LSD1 are GTCGGACCAGCCGGCGCAAG (sgRNA #1) and CGCGGAGGCTCTTTCTTGCG (sgRNA #2). Stably transfected cells were isolated using puromycin selection, followed by single‐cell clone selection. After limiting dilution and expanding cultivation, LSD1 expression was detected by western blot to evaluate the knockout effect.

### 4.15 Western blot analysis

The western blot analysis was performed as described previously.^21^ Briefly, cells were lysed by using radioimmunoprecipitation (RIPA) lysis buffer with protease and phosphatase inhibitors A/B (Selleckchem). The total protein levels were assessed through G250 (Bio‐Rad). Protein was electrophoresed and isolated by tris‐HCl gel after denaturation and transferred to poly‐vinylidene fluoride membranes (Millipore). Following 5% skimmed milk blocking at 25°C, loaded membranes were placed in incubation with specific antibodies at 4°C overnight. The essential antibody information is listed below: Cyclin A2 (CST, #4656), CDK6 (CST, #3136), CDK4 (CST, #12790), Cyclin E (CST, #4129), β‐actin (CST, #3077), LSD1 (CST, #2139), H3K4me1 (CST, #5326), H3K4me2 (CST, #9725), H3K9me1 (CST, #14186), and H3K9me2 (CST, #4658). Membranes were consequently thrice‐washed using tris‐HCI buffer solution with tween 20 (TBST) and incubated with horseradish peroxidase (HRP)‐conjugated secondary antibodies at 37°C for 1 h. Amplified protein markings were identified through chemiluminescence imaging after incubation with chemiluminescent HRP substrate (Millipore).

### 4.16 RT‐qPCR

Total RNA was freshly purified from cells with Total Miniprep Kit (Axygen). The cDNA was prepared with PrimeScript RT reagent Kit (Takara). Consequently, cDNA quantification was performed using the CFX RT‐PCR detection system (Bio‐Rad, Hercules, USA) with SYBR Green SuperMix (Bio‐Rad). Primer information is listed in the Supplementary Information (Table S6). All samples were tested in triplicate and relative expression levels were normalized to *ACTIN*.

### 4.17 mRNA‐seq and bioinformatics

mRNA‐seq was performed through a profiler service (NOVOGENE). Total RNA was purified with TRIzol (Invitrogen) from HepG2 and Hep3B cells, which were exposed to ZY0511 (2 μM) for 48 h. Triplicate samples were used per group, with relevant probe sets filtered for detection utilizing fold‐change > 1.5, *p* < 0.05 (Student's *t*‐test), and false discovery rate < 0.05. The enriched pathway analysis was carried out in the KEGG, using standard settings. GSEA was performed with the LSD1 downstream gene sets (http://cistrome.org/CistromeCancer/), using the GSEA software (Broad Institute, version v2.2.0).

### 4.18 siRNA library screening

The siRNA library screening was conducted using a customized siRNA library against the selected 10 genes (RiboBio). Each mRNA was targeted by an siRNA pool (50 nM) consisting of three genOFF oligonucleotides (RiboBio). HepG2 cells were seeded into plates (6 × 10^3^ cells/well) and then transfected with siRNA in Lipofectamine 3000 for 16 h. The proliferation of HepG2 cells was detected by MTT assay 72 h after transfection. The function of target genes was identified based on cell proliferation, compared with the control.

### 4.19 ChIP assay

ChIP was carried out on HCC cells incubated with 2 μM ZY0511 in line with the chromatin immunoprecipitation Kit protocol (Millipore, #17‐10086). In detail, cells were fixated using 1% formaldehyde. Cross‐linked chromatin was sheared with a sonifier to reveal 200–500 bp DNA strands. The lysate was pre‐cleared using protein A/G agarose, followed by incubating with targeting antibodies, including anti‐LSD1 (Millipore, #17‐10531), anti‐H3K4me1 (CST, #5326), anti‐H3K4me2 (CST, #9725) or IgG (Millipore, #17‐10086), at 4°C overnight. The protein/DNA complexes were collected, and enriched DNA was quantified using RT‐qPCR.

### 4.20 Immunofluorescence

HCC cells were seeded on coverslips and treated with ZY0511 or SP2509 for 48 h, followed by fixing with 4% paraformaldehyde for 15 min. Consequently, cells were permeabilized with PBS containing 0.2% Triton X‐100 for 20 min at 25°C. Following blocking with PBST containing 0.2% bovine serum albumin (BSA) for 60 min, permeabilized cells were incubated with primary antibodies of GADD45B (Abcam, ab105060) at 4°C overnight and incubated with the cy3‐conjugated secondary antibodies for 60 min at room temperature. The slides were stained with 4', 6‐diamidino‐2‐phenylindole (DAPI) for nuclear detection. The IF images were acquired with DeltaVision Ultra‐high resolution microscope (GE Healthcare) with a 63 × NA oil objective.

### 4.21 CDX and For CDX models, HCC cells (HepG2, Hep3B) PDX models

For CDX models, HCC cells (HepG2, Hep3B) were harvested, and cells suspension (5 × 10^6^ − 1 × 10^7^ cells per mouse) were subcutaneously injected into the hind flank region of nude mice. For PDX models, fresh HCC tissues obtained from West China Hospital were minced into 2–5 mm^3^ sizes to build the PDX models. The HCC tissue fragments were transplanted into the hind flank region of NOD/SCID mice within 2 h. Once the tumor volume reached ∼2 cm^3^, the PDX models were expanded.

When the tumor volume reached approximately 100 mm^3^, CDX & PDX tumor‐bearing mice were randomly divided into groups: (1) blank solvent; (2) ZY0511 50 mg/kg per day, i.p.; (3) ZY0511 100 mg/kg per day, i.p.; and (4) combinatorial treatment of ZY0511 50 mg/kg with DTP3 30 mg/kg, per day, i.p. Tumor sizes and animal body weights were monitored every 3 days, and tumor volumes were calculated as 0.5 × length × width^2^. TGI rate was calculated as (1 − tumor volume of treated group/tumor volume of control group) x 100%.

### 4.22 H&E and IHC staining

Both procedures were carried out as previously described.^21^ In brief, samples were fixated in 4% paraformaldehyde and consequently paraffin‐coated. Thin tissue segments (4 μm) were prepared, and H&E staining was performed. For IHC, following heat antigen retrieval, the tumor segments were incubated with primary antibodies at a recommended dilution concentration, including Ki67 (Abcam, ab16667), PCNA (Servicebio, GB11010), Cleaved caspase 3 (CST, #9664), LSD1 (CST, #2139), H3K4me1 (CST, #5326), H3K4me2 (CST, #9725), and GADD45B (Abcam, ab105060). Consequently, the slides were incubated with biotinylated secondary antibody, visualized with 3,3′‐diamino‐benzidine substrate, and photographed using an optical microscope (Olympus). Relative protein expression assessment was attained through the integral optical density (IOD) and area, collected by Image J software, together with average optical density (AOD) calculation. The AOD reflected targeted proteomic expression per unit area.

### 4.23 Acute toxicity assays

To test the safety profile of ZY0511 and SP2509, acute toxicity assays were performed. In detail, the mice were intraperitoneally administrated ZY0511 or SP2509 (400 mg/kg) within 24 h. On the 14th day, the mice were sacrificed. The major organs were collected for H&E staining assay. The blood was collected for blood routine tests and biochemical tests.

### 4.24 Statistical analysis

Data are presented as mean ± SD or SEM. Pearson's correlation test was used for correlation analysis, and the log‐rank test was used for survival rate analysis. Statistical analyses were conducted by GraphPad Prism 8 software. The unpaired, two‐tailed *t‐*test was employed to determine significant differences in the data between two experimental groups, and one‐way analysis of variance was used to compare the three (or more) un‐matched groups. All statistical analyses were two‐sided; ^*^ *p* < 0.05, ^**^ *p* < 0.01, ^***^ *p* < 0.001, and ^****^ *p* < 0.0001 were considered significant.

## AUTHOR CONTRIBUTIONS

Y.L.Z. and N.S. designed the study and wrote the manuscript. N.S. and X.Z. conducted the major experiments. K.G., H.L., and J.X. took part in some major experiments. Y.Z., X.Z., Y.Z.L., R.L., L.Y.Z., L.F., J.Y.Z., D.L., and C.L. helped to conduct part of in vitro experiments. Q.X.S., J.L., S.Y.Y., and X.B.C. gave indispensable guidance to the whole study and manuscript. Z.Q.C., Y.L., L.T., N.S., J.H.Q., X.Y.Y., and Z.P.Z. gave some constructive advises of experiments. All authors read and approved the submitted manuscript.

## CONFLICT OF INTEREST STATEMENT

The authors declare no conflicts of interest.

## ETHICS STATEMENT

Building PDX models were conducted with the approval of West China Hospital (Ethics Number: 20191025). The tissue chip of human HCC tissues was approved by the National Engineering Center for Biochips (Ethics Number: YB M‐05‐02). Informed consent was obtained from participants or their guardians. All animal experiments were approved by the Institutional Animal Care and Treatment Committee of Sichuan University (Permit Number: 2019030502) and were performed in conformity with the ARRIVE guidelines.

## Supporting information

Supporting Information

## Data Availability

All data supporting the findings of this study are available from the corresponding author upon reasonable request.
