# Supplementary material for: Pharmacological inhibition of LSD1 suppresses growth of hepatocellular carcinoma by inducing GADD45B
Source: MedComm (2020). 2023 May 24;4(3):e269. doi: 10.1002/mco2.269 (PMC10209615; doi:10.1002/mco2.269)
Supplement: Supplementary file 1 — Supporting Information [file MCO2-4-e269-s001.pdf]

Pharmacological inhibition of LSD1 suppresses growth of hepatocellular carcinoma by inducing GADD45B

Na Sang[1, 4, #], Xi Zhong[2, #], Kun Gou[1], Huan Liu[2, 3], Jing Xu[1], Yang Zhou[1], Xia Zhou[1], Yuanzhi Liu[1], Zhiqian Chen[2], Yue Zhou[1], Yan Li[1], Lei Tao[1], Na Su[5], Lingyun Zhou[6], Jiahao Qiu[1], Xinyu Yang[2], Zeping Zuo[1], Li Fu[7], Jingyao Zhang[7], Dan Li[7], Cong Li[7], Qingxiang Sun[1], Jian Lei[1], Rui Li[1], Shengyong Yang[1], Xiaobo Cen[3], Yinglan Zhao[1, 2]

[1] Department of Biotherapy, Cancer Center and State Key Laboratory of Biotherapy, West China Hospital, West China Medical School, Sichuan University, Chengdu, 610041, China

[2] Department of Pharmacology, Key Laboratory of Drug Targeting and Drug Delivery System of the Education Ministry, Sichuan Engineering Laboratory for Plant-Sourced Drug and Sichuan Research Center for Drug Precision Industrial Technology, West China School of Pharmacy, Sichuan University, Chengdu, 610041, China

[3] National Chengdu Center for Safety Evaluation of Drugs, State Key Laboratory of Biotherapy, West China Hospital, Sichuan University, Chengdu, 610041, China

[4] Department of Radiation Oncology, Radiation Oncology Key Laboratory of Sichuan Province, Sichuan Clinical Research Center for Cancer, Sichuan Cancer Hospital & Institute, Sichuan Cancer Center, Affiliated Cancer Hospital of University of Electronic Science and Technology of China, Chengdu, China

[5] Department of Pharmacy, West China Hospital, West China Medical School, Sichuan University, Chengdu, 610041, China

[6] Center of Infectious Diseases, West China Hospital, Sichuan University, Chengdu, 610041, China

[7] Core Facility Center, West China Hospital, Sichuan University, Chengdu, 610041, China

Correspondence

Yinglan Zhao, Department of Biotherapy, Cancer Center and State Key Laboratory of Biotherapy, West China Hospital, West China Medical School, Sichuan University, 17#, 3rd Section, Ren min South Road, Chengdu 610041, China.

E-mail: zhaoyinglan@scu.edu.cn

Na Sang and Xi Zhong contributed equally to this work.

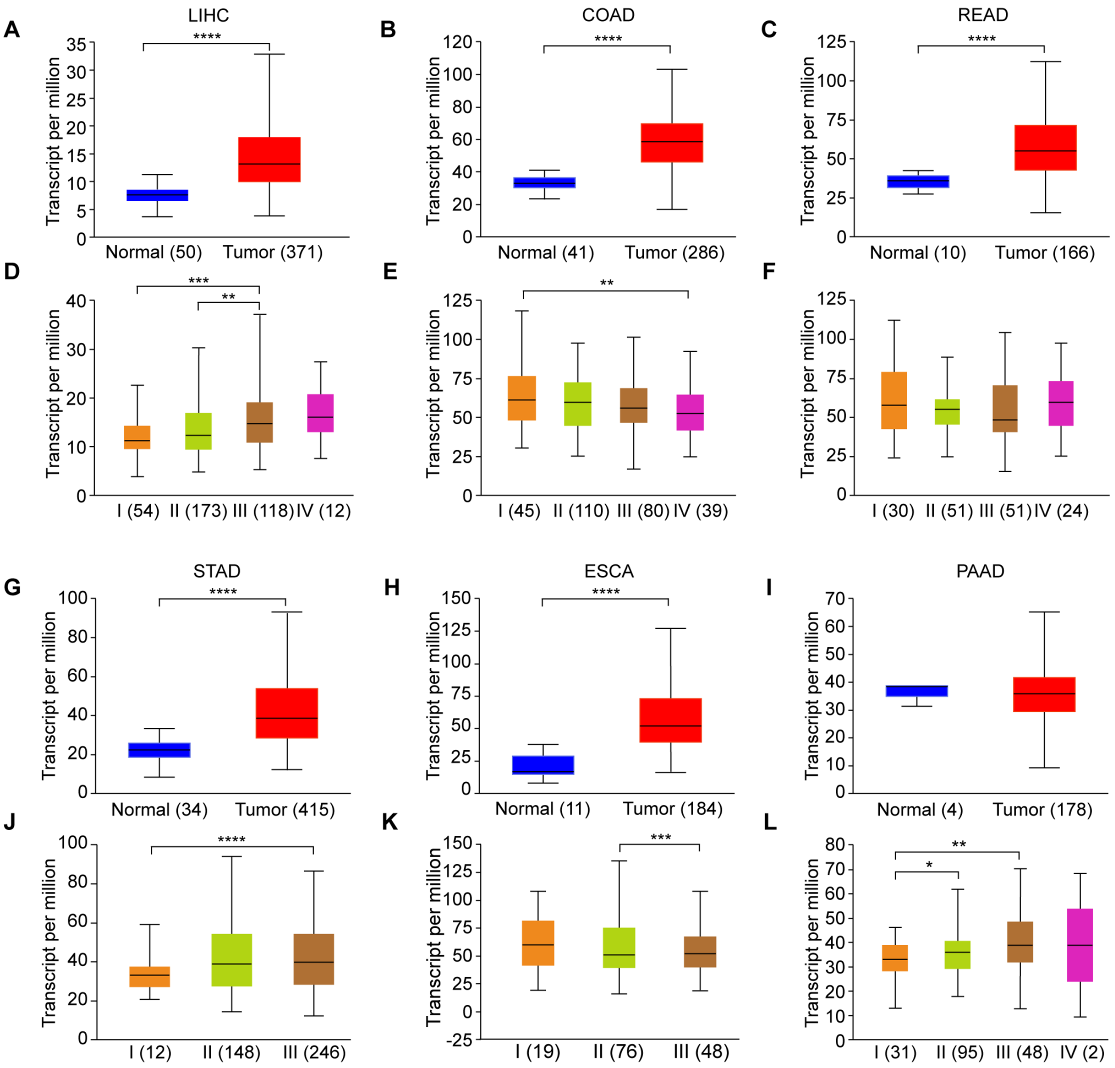

Figure S1 *KDM1A* is overexpressed in human HCC tissues and inversely correlates with patients' progression

The gene expression of *KDM1A* in cancer tissues and normal adjacent tissues (NATs), and the correlation of *KDM1A* with tumor grade in six gastrointestinal cancers including LIHC (A, D), COAD (B, E), READ (C, F), STAD (G, J), ESCA (H, K) and PAAD (I, L) was analyzed by using UALCAN website. Data are presented as mean ± SD. *$P < 0.05$; **$P < 0.01$; ***$P < 0.001$; ****$P < 0.0001$.

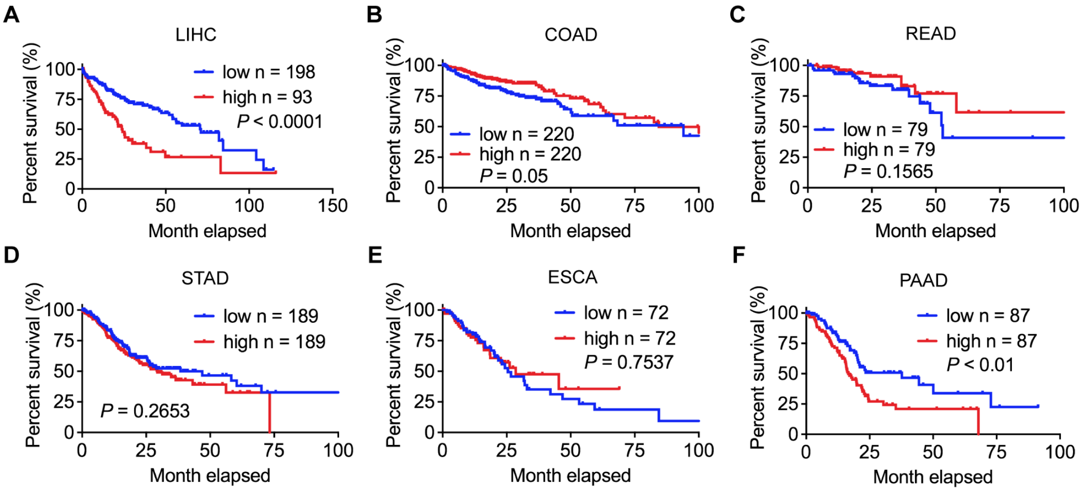

Figure S2 The overexpression of *KDM1A* is correlated with the poor survival of HCC patients

Correlation analysis of *KDM1A* expression and patient survival was performed by Kaplan-Meier analysis in six gastrointestinal cancer patients including LIHC (A), COAD (B), READ (C), STAD (D), ESCA (E) and PAAD (F) using OncoLnc website.

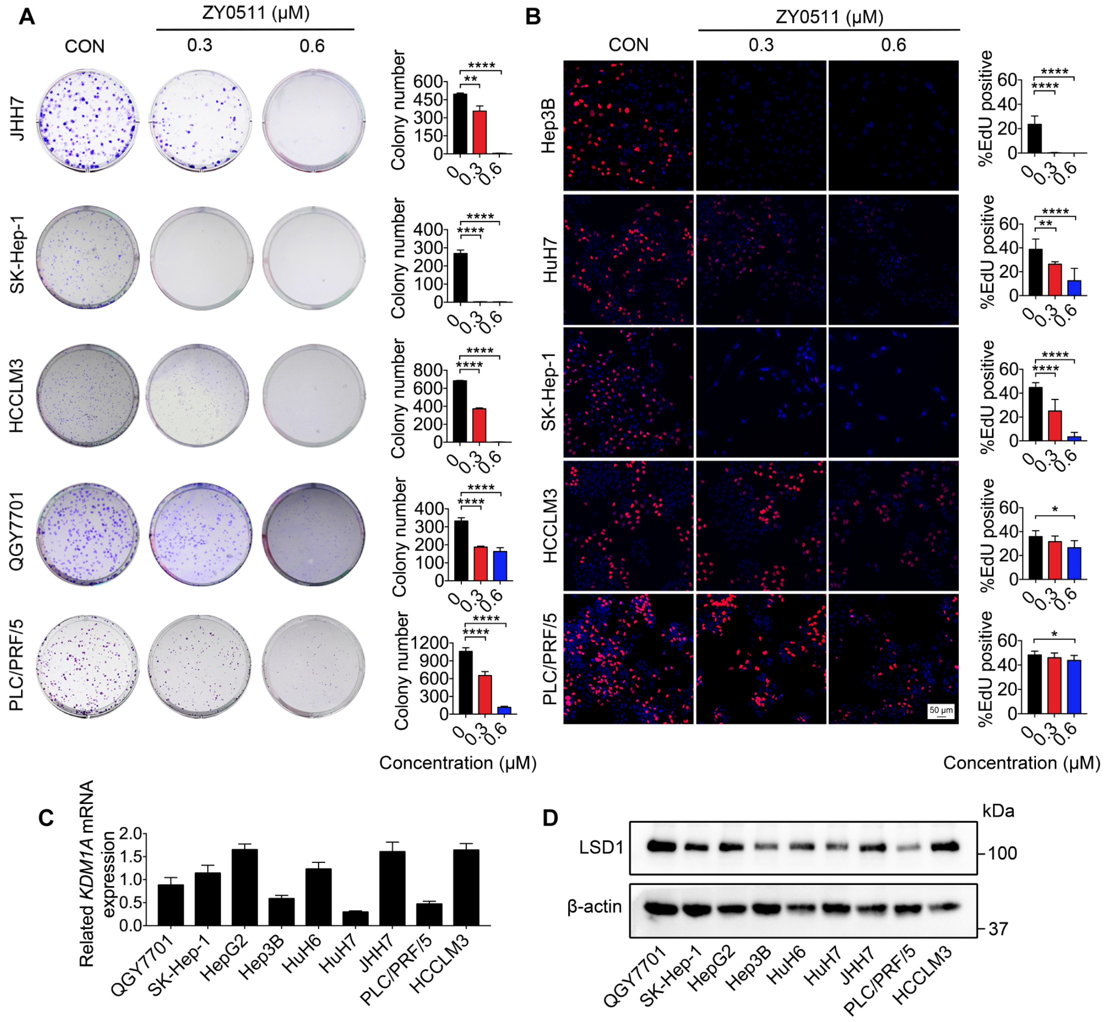

Figure S3 ZY0511 inhibits HCC cells proliferation *in vitro*

(A) Representative images (left) and quantification (right) of colony formation assay in HCC cells post-exposure to different concentrations of ZY0511 for 10-14 days (n = 3). (B) Representative images (left) and quantification (right) of EdU incorporation assay in HCC cells post-exposure to different concentrations of ZY0511 for 96 h (red: EdU; blue: Nucleus) (n = 3). Scale bar, 50 μm. (C) The mRNA levels of *KDM1A* were determined by RT-qPCR in nine HCC cells (n = 3). (D) The expression of LSD1 protein in nine HCC cells was determined by western blot analysis. β-actin was the reference

protein. Data are presented as mean ± SD. *$P < 0.05$, **$P < 0.01$, ****$P < 0.0001$ vs control.

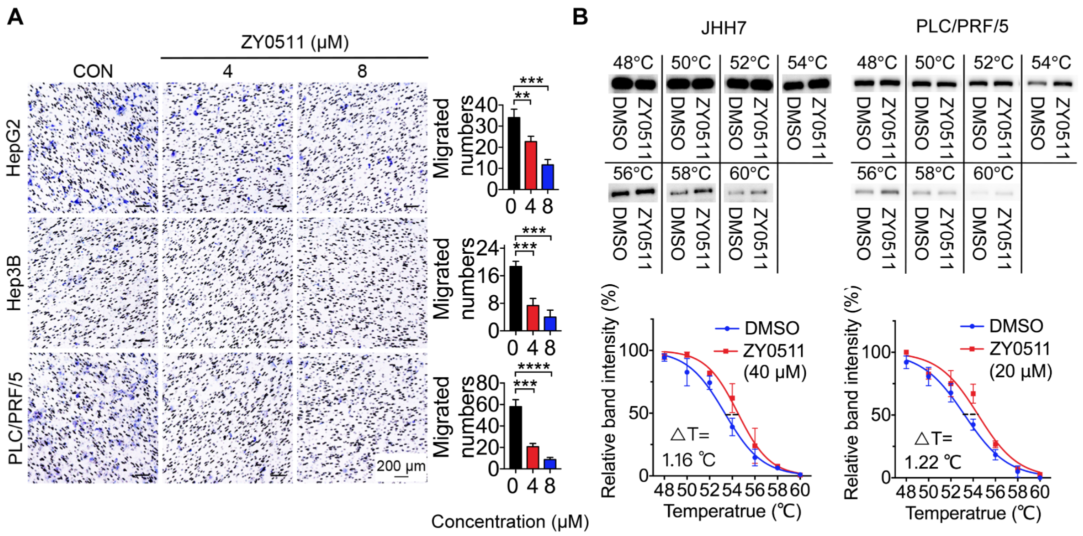

Figure S4 ZY0511 inhibits migration of HCC cells *in vitro*

(A) Representative images (left) and quantification (right) of transwell assay in HCC cells post-exposure to different concentrations of ZY0511 for 48 h ($n = 3$). Scale bar, 200 μm. (B) Representative images (above) and quantification (below) of CETSA melt curve from 48 ℃ to 60 ℃ of HCC cells lysates with DMSO or ZY0511 incubation ($n = 3$). Data are presented as mean ± SD. **$P < 0.01$, ***$P < 0.001$, ****$P < 0.0001$ vs control.

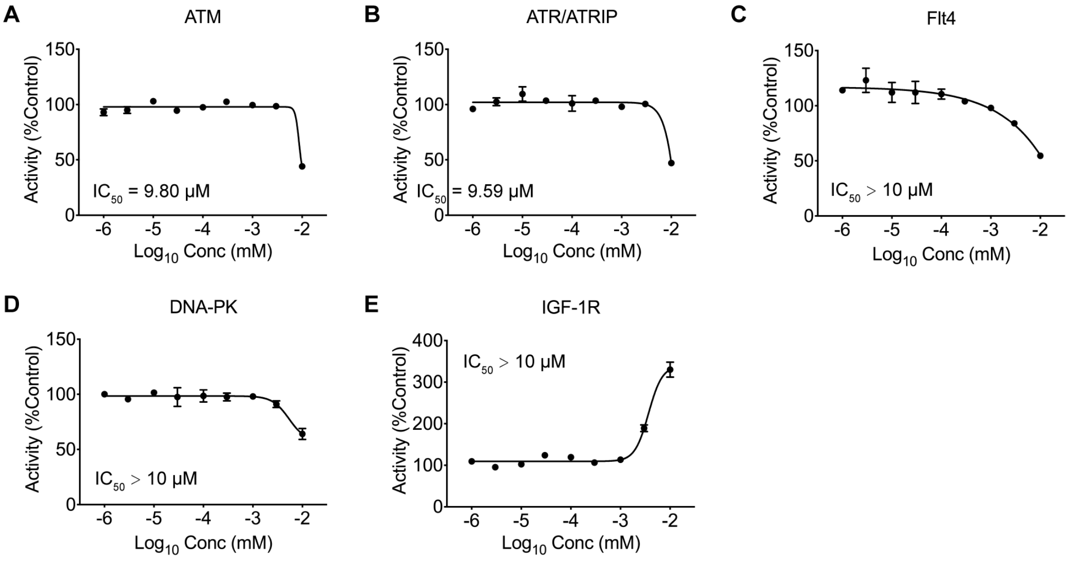

Figure S5 ZY0511 does not inhibit activity of ATM, Flt4, ATR/ATRIP, DNA-PK and IGF-1R

(A-E) The effect of ZY0511 on ATM (A), ATR/ATRIP (B), Flt4 (C), DNA-PK (D) and IGF-1R (E) activity was analyzed and IC_{50} values were calculated (n = 3).

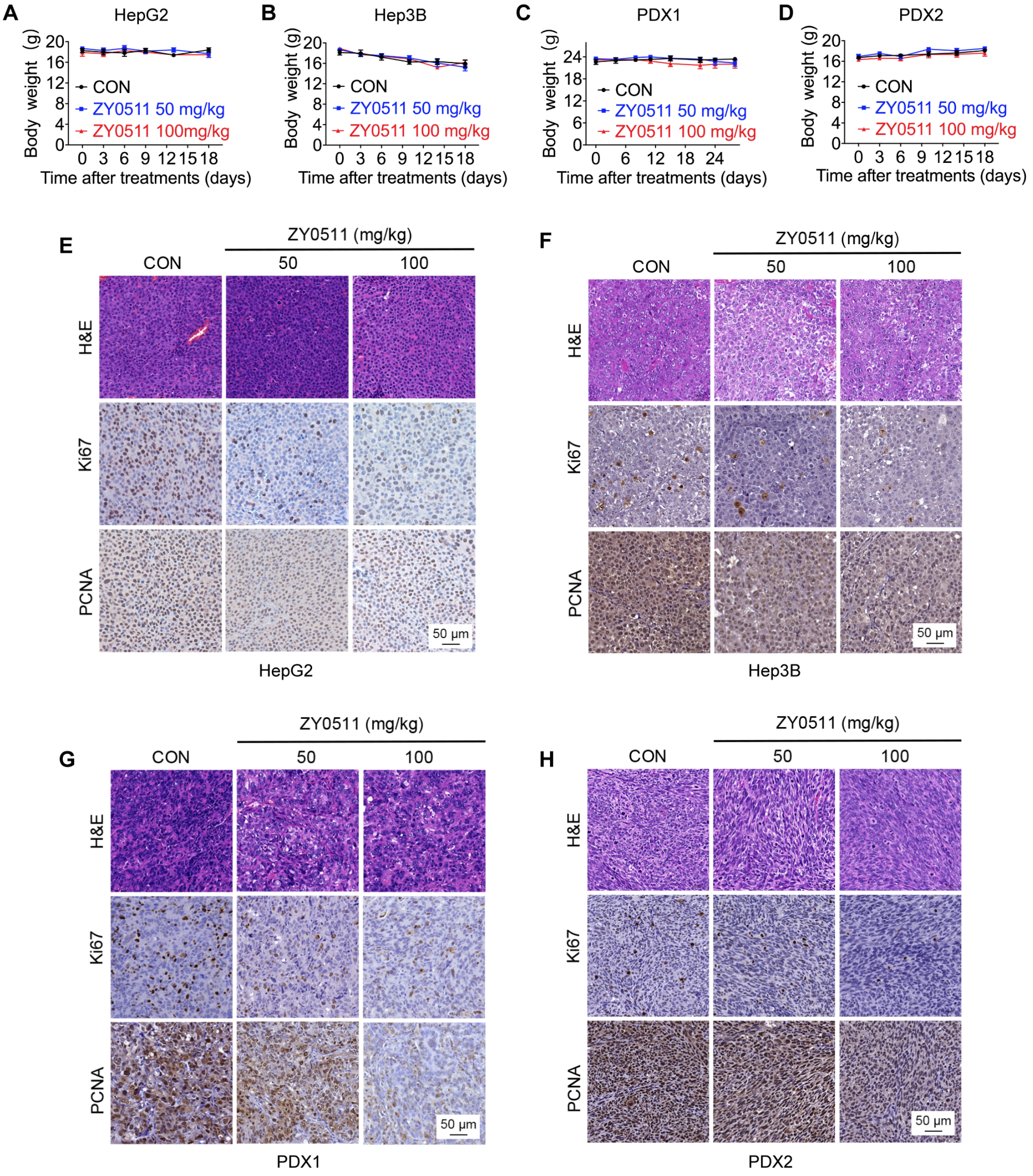

Figure S6 ZY0511 reduces the expression of Ki67 and PCNA in subcutaneous tumor tissues

(A-D) Body weight curve of mice with HepG2 (A), Hep3B (B), PDX1 (C) and PDX2 (D) subcutaneous tumors post-ZY0511 treatment. *n* = 6 mice per group. (E-H) Representative images of hematoxylin & eosin and immunohistochemistry staining of

Ki67 and PCNA on HepG2 (E), Hep3B (F), PDX1 (G) and PDX2 (H) subcutaneous tumors post-ZY0511 administration. Scale bar, 50 μm.

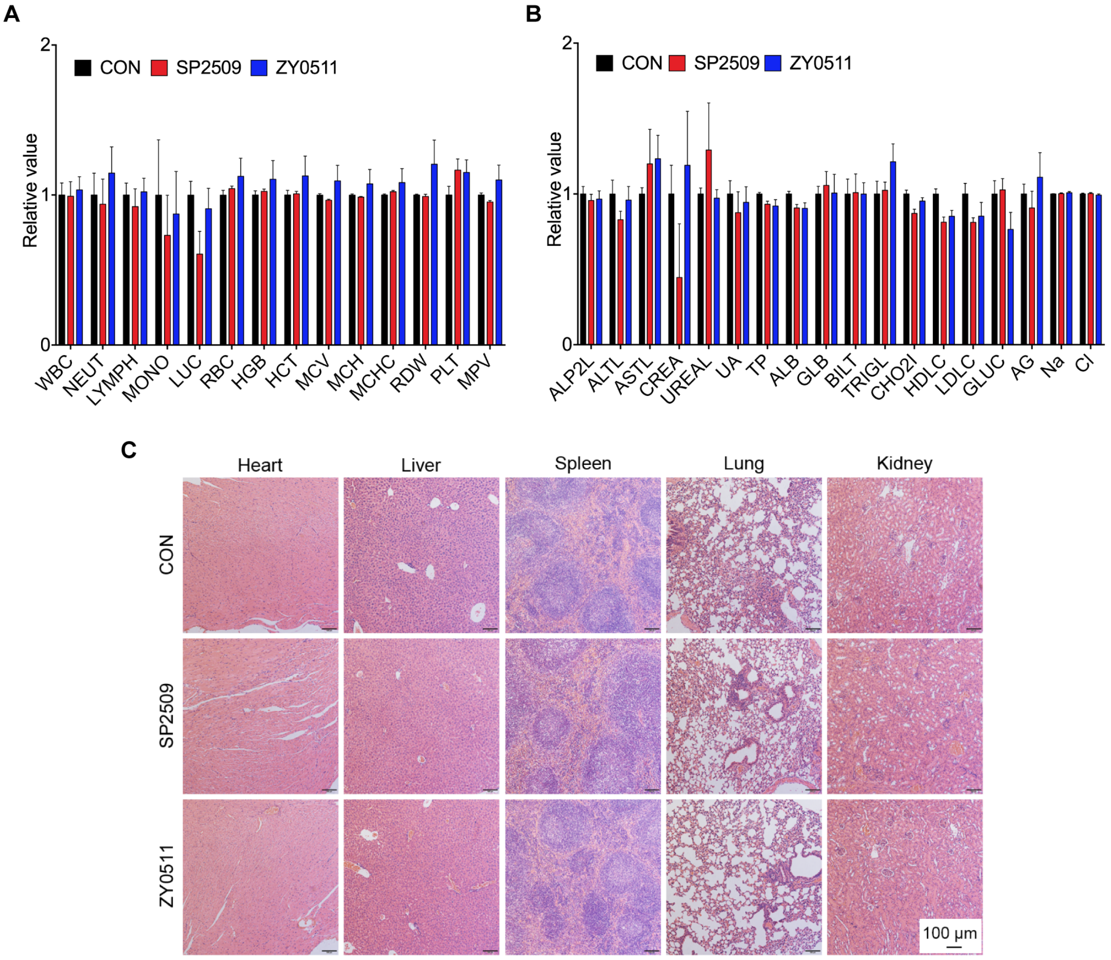

Figure S7 ZY0511 is well tolerated *in vivo*

(A-B) The parameters of blood routine tests (A) and biochemical tests (B) from mice in the acute toxicity assay. n = 8 mice per group. (C) Representative hematoxylin & eosin staining images of the main organs in mice with ZY0511 and SP2509 treatment in the acute toxicity assay. Scale bar, 100 μm. Data are presented as mean ± SEM.

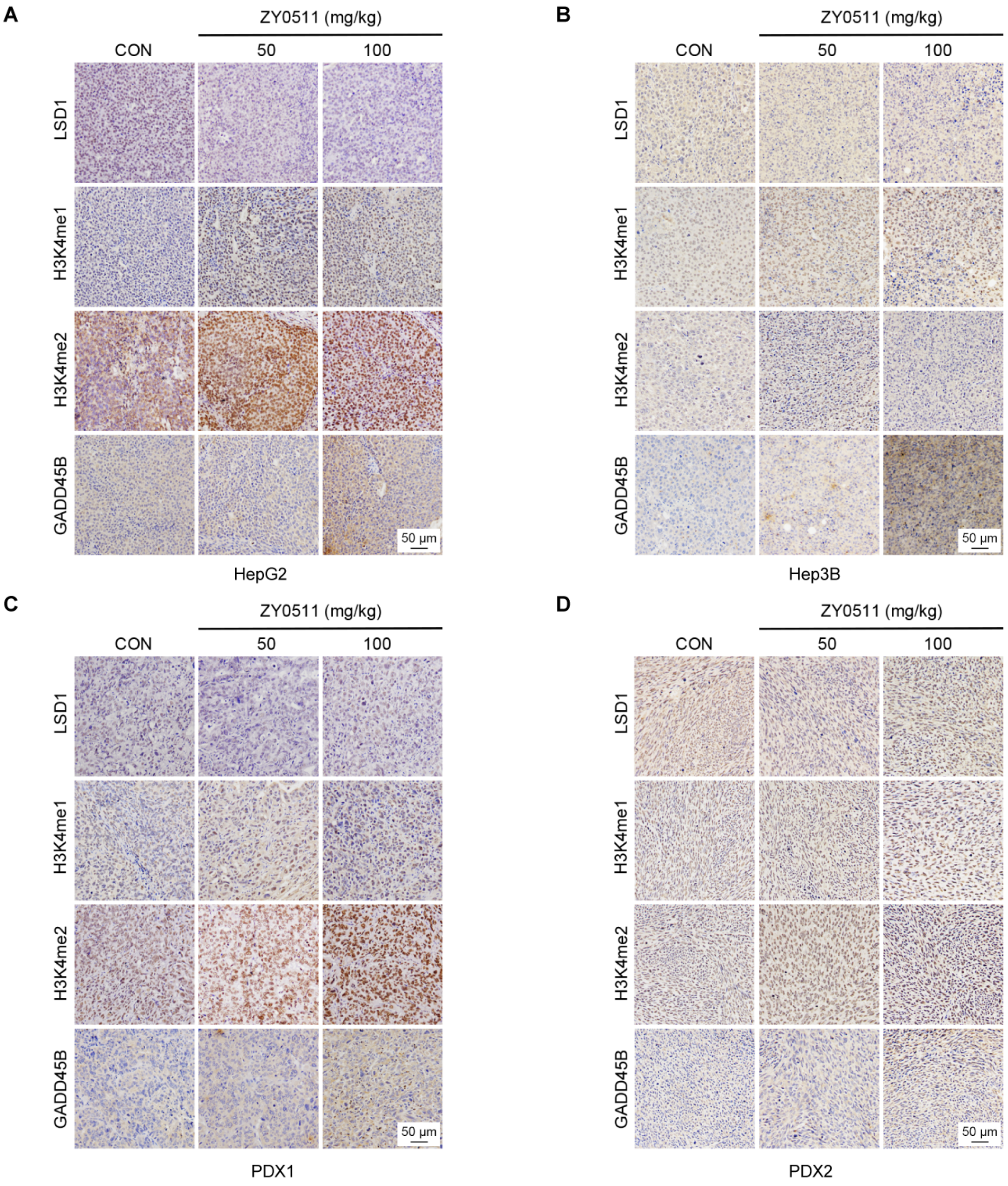

Figure S8 ZY0511 increases the expression of H3K4me1/2 and GADD45B instead of LSD1 in subcutaneous tumor tissues

(A-D) Representative images of immunohistochemistry staining of LSD1, H3K4me1/2 and GADD45B on HepG2 (A), Hep3B (B), PDX1 (C) and PDX2 (D) subcutaneous tumors post ZY0511 administration. Scale bar, 50 μm.

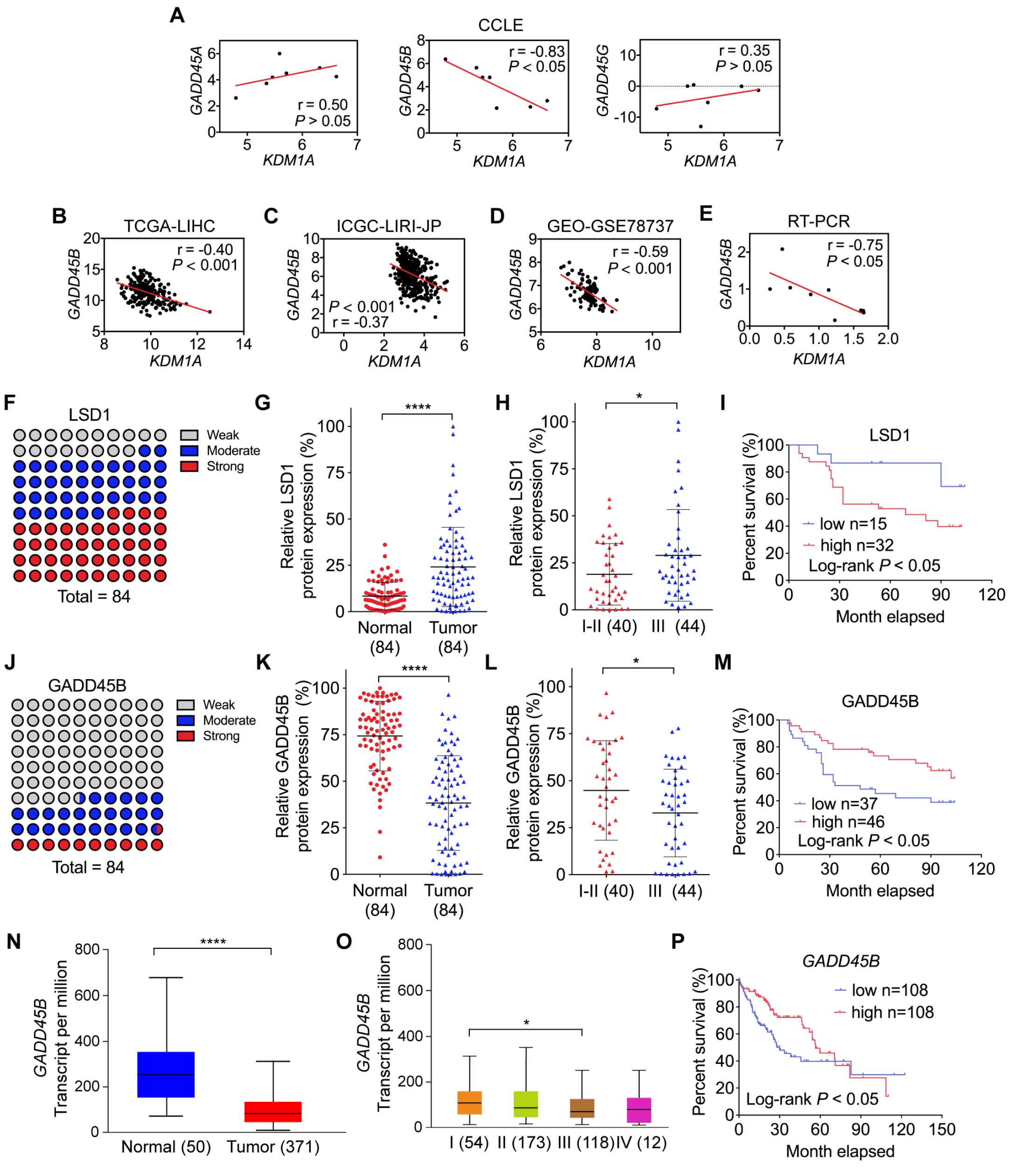

Figure S9 The LSD1 level inversely correlates with GADD45B level in human HCC tissues

(A) Correlation between mRNA levels of *GADD45A*, *GADD45B* and *GADD45G* and *KDM1A* in HCC cells using CCLE datasets. (B-D) Correlation between mRNA levels of *GADD45B* and *KDM1A* in HCC tissues using TCGA (B), ICGC (C) and GEO (D) databases. (E) Correlation between the mRNA levels of *GADD45B* and *KDM1A* in

eight HCC cells using RT-qPCR results. (F) The percentage of strong, medium and weak LSD1 level in HCC tissues. (G) The expression of LSD1 in HCC tissues and NATs. (H) The expression of LSD1 in HCC tissues with different pathological grades. (I) Kaplan-Meier survival curves for LSD1 expression in HCC patients. (J) The percentage of strong, medium and weak GADD45B level in HCC tissues. (K) GADD45B level in HCC tissues and NATs. (L) The expression of GADD45B in HCC tissues with different pathological grades. (M) Kaplan-Meier survival curves for GADD45B expression in HCC patients. (N) The gene expression of *GADD45B* in HCC tissues and NATs was analyzed by using UALCAN website. (O) The gene expression of *GADD45B* in HCC tissues with different pathological grades was analyzed by using UALCAN website. (P) Kaplan-Meier survival curves for *GADD45B* expression in HCC patients was analyzed by using OncoLnc website. Data are presented as mean ± SD. *$P < 0.05$, ****$P < 0.0001$.

Table S1 Proliferation inhibitory effect of LSD1 inhibitors against HCC cells for 144 h *in vitro*

Cell line	IC_{50} (μM)	
	GSK2879552	ORY1001
HepG2	>100	>100
Hep3B	>100	28.57±6.72
HuH6	>100	46.26±1.39
JHH7	>100	56.23±9.07
HuH7	>100	>100
SK-Hep-1	>100	35.81±5.05
HCCLM3	>100	>100
QGY7701	>100	>100
PLC/PRF/5	>100	>100

Table S2 The inhibition effect of ZY0511 (1 μM) against cancer-related kinases

Kinase	Inhibition rate (%)	Kinase	Inhibition rate (%)
ATM	75	PI3 Kinase (p120γ)	10
Flt4	62	CDK5/p35	9
ATR/ATRIP	62	EGFR (L858R)	8
DNA-PK	60	Flt3	8
B-Raf (V599E)	51	PDK1	8
Tie2	50	PKCε	8
Fer	47	PI3 Kinase (p110βγ/p85α)	8
c-RAF	43	cKit (V560G)	7
MST3	35	EGFR	7
KDR	30	Fms	7
Blk	29	TrkC	7
B-Raf	29	EGFR (L861Q)	6
Hck	29	HIPK3	6
Fes	24	MEK1	6
CK1γ3	21	ZIPK	6
FGFR3	20	PI3 Kinase (p110δ/p85α)	6
Flt1	20	PI3 Kinase (p110α/p85α)	6
Met	20	PI3 Kinase (p110α (H1047R)/p85α)	6
PAK4	20	DDR2	5
Aurora-A	17	DYRK2	5
Bmx	17	BRK	4
LKB1	15	CSK	4
Pim-1	15	PKBα	4
PI3 Kinase (p110α (E545K) /p85α)	15	Snk	4

FGFR4	14	DCAMKL3	3
Abl	13	FAK	3
CK1γ2	13	PKCθ	3
HIPK2	13	EphA7	2
CK1γ1	12	NIM1	2
FGFR2	11	PKCι	2
Flt3 (D835Y)	11	PKD2	2
p70S6K	11	PI3 Kinase (p110α (E542K)/p85α)	2
ROCK-I	11	Abl (T315I)	1
Ros	11	MELK	1
CDK2/cyclinA	10	TAK1	1
PKCμ	10	Yes	0

Table S3 The activation effect of ZY0511 (1 μM) against cancer-related kinases

Kinase	Activation rate (%)	Kinase	Activation rate (%)
IGF-1R	185	cSRC	4
PDGFRα	33	PKCα	4
ErbB2	25	ZAP-70	4
ALK	23	Axl	3
MAPK2	16	CDK1/CyclinB	3
Ret	15	TrkA	3
cKit	14	CHK2	2
IKKα	13	cKit (D816H)	2
Wee1	11	EGFR (T790M, L858R)	2
ErbB4	10	Mer	2
Ron	7	PDGFRα (D842V)	2
ARK5	6	PKCη	2
DAPK1	5	CHK1	1
EGFR (T790M)	5	FGFR1	1
EphA2	5	IKKε	1
EphB4	5	PAK1	1

Table S4 Characteristics of the selected genes

Gene	Correlation with KDM1A (r)	COX	Fold change (HepG2)	Fold change (Hep3B)
GADD45B	-0.319	-0.259	9.57	3.34
ANKZF1	0.287	0.18	1.28	5.67
BCL10	0.121	0.259	6.33	1.51
CDC42	0.486	0.228	6.21	1.88
CDKN2D	0.206	0.209	2.80	2.56
CEP250	0.333	0.208	1.97	2.87
E2F7	0.249	0.266	4.24	2.83
EDEM1	-0.245	-0.163	1.74	1.76
EGLN3	0.188	0.359	34.47	12.95
FBXL5	-0.186	-0.31	2.08	1.61
GAPDH	0.234	0.212	2.47	2.14
JMJD6	0.207	0.258	3.16	1.30
KIAA0355	-0.123	-0.144	2.53	2.37
KLHL15	-0.172	-0.107	4.60	1.71
NARF	0.278	0.113	2.44	2.21
PGM2L1	0.107	0.242	3.84	5.18
RIT1	0.129	0.379	5.58	3.26
SEMA3G	-0.258	-0.214	3.70	23.44
SLC25A4	-0.127	-0.295	1.59	2.17
SLC4A11	0.139	0.156	4.20	1.63
TAPT1	-0.358	-0.373	1.20	2.42
TCTA	-0.131	-0.129	2.17	1.48
TFRC	0.053	0.12	3.17	1.70
TMCC1	0.162	0.251	4.47	5.21
TUBA4A	0.0661	0.258	2.41	5.07
UBE2D1	0.135	0.278	2.75	1.35

UPP1	0.112	0.168	2.68	4.92
ZSWIM5	0.414	0.263	1.23	2.39

Table S5 Clinical characteristics of patients in tissue chip

Characteristics	n (%)
**Gender**	
Male	76 (90%)
Female	8 (10%)
**Age (yr)**	
⩾50	49 (58%)
<50	35 (42%)
**Tumor capsule**	
Complete	45 (54%)
Incomplete	38 (45%)
Unkown	1 (1%)
**Vascular invasion**	
Yes	21 (25%)
No	55 (65%)
Unkown	8 (10%)
**Cirrhosis**	
Yes	75 (89%)
No	8 (10%)
Unkown	1 (1%)

Table S6 Primer sequences in RT-qPCR

Gene	Forward primers	Reverse primers
ACTIN	CATGTACGTTGCTATCCAGGC	CTCCTTAATGTCACGCACGAT
ANKZF1	TGGAGGCACGGCAGGCTAC	GTCACTGAGGGCGGCAAATCG
BCL10	GAGCCACGAACAACCTCTCCAG	AAAGGGCGTCGTGCTGGATTC
CDC42	GAAAAGCTGGCCCGTGACCTG	CTGCGGCTCTTCTTCGGTTCTG
CDKN2D	CAAGGTGCCAGCCCCAATGTC	AGGGTGTCCAGGAATCCAGTGC
CEP250	GCTGCTGGCATCCTGGAAGAAG	TGGGCTCTCTCACGCTCACTC
E2F7	CGTCAGGGTCAGGGTCAGAGAG	AGTGGCTGGCTCATCCTCCTC
EDEM1	ACGAGCAGTGAAAGCCCTTTGG	CCAGTGGCCCGTCTGAATGTTC
EGLN3	CCTGTCTGCACGAGGCAATGG	CCCACCATGTAGCTTGGCATCC
FBXL5	GGGCTGCCTTATTTGGAGCA	GGCGGTATCAGCATGAGGAC
GAPDH	CAGGAGGCATTGCTGATGAT	GAAGGCTGGGGCTCATTT
GADD45B	CCAGTCCTTCTGCTGTGACAACG	TCTTCCAGGCGTCCGTGTGAG
JMJD6	CGCAGACTCGGTTGACCTTCAG	TGACGAACTGGAGCTGGAGGAG
KIAA0355	GGCGTGAGAACTTCCTGCATGG	ACGGAGAGCCCTGATGTGACTG
KLHL15	TGAAGTCAAGCCGCATCCGTTC	GGCCTTCTAGCTCCCACCAGAC
NARF	CGGGCACCTGGCACACATC	GGGCTCGGTAAGTGACCTCCTC
PGM2L1	ATGGCTGTGTTGCTACCCTTCG	GGTCAGGTGACGCACACATCTC
RIT1	AGCCACCGATTCCCAGAAGA	GTCCCGCATGGCTGTAAACT
SEMA3G	GCCTGGACGCCCTCTACTCTC	GTAGCACCCGCACGAAGTTGG
SLC25A4	TCTACCCGCTGGACTTTGCT	GCCCAGACCATGGAACTCAC
SLC4A11	GGTTCGGTTCGTCATCCTGGTG	ATTCCTCCTCTGTGCGGGTCTC
TAPT1	TCGTGCTGTTGGGGAAATCGTG	TACTGGACGGCTTGCCTGGAG
TCTA	GGGTTGTATCACCGTCCAGG	TTGCTGCCATTTCCCACGAA
TFRC	AGGGAGGAGCCAGGAGAGGAC	GATGGTGCCGGTGAAGTCTGTG
TMCC1	TCCTGGCTGTCATGGCAGTCC	GCTGAACGTCCTGTTGCGAGTC
TUBA4A	TGCCTGCTGGGAGCTCTATT	CCGGGGTACGTGTTTTCCAG
UBE2D1	AGCGCATATCAAGGTGGAGTC	TGTTGTGAAAGCAATCTTTGGTGG
UPP1	CGGTGCTGTCTGTCAGTCATGG	ACCAGAAGTGCCAATGCGGATG
ZSWIM5	GCCGCCGAAGCTCTGGAAAG	GCGAGGGCTGATGTGTGTTAGG
